# *Cis*-regulatory effects of carrot miniature inverted-repeat transposable elements on the expression of genes controlled by LHY/RVE transcription factors

**DOI:** 10.1093/hr/uhaf360

**Published:** 2026-01-02

**Authors:** Alicja Macko-Podgórni, Kinga Zygmuntowicz, Wojciech Wesołowski, Kornelia Kwolek, Marcelina Skrabucha, Emilia Data, Zuzanna Boczar, Zara Maria Lacera, Charles Nathan Hancock, Dariusz Grzebelus

**Affiliations:** Department of Plant Biology and Biotechnology, University of Agriculture in Krakow, al. 29 Listopada 54, Krakow, Poland; Department of Plant Biology and Biotechnology, University of Agriculture in Krakow, al. 29 Listopada 54, Krakow, Poland; Department of Plant Biology and Biotechnology, University of Agriculture in Krakow, al. 29 Listopada 54, Krakow, Poland; Department of Plant Biology and Biotechnology, University of Agriculture in Krakow, al. 29 Listopada 54, Krakow, Poland; Department of Plant Biology and Biotechnology, University of Agriculture in Krakow, al. 29 Listopada 54, Krakow, Poland; Department of Plant Biology and Biotechnology, University of Agriculture in Krakow, al. 29 Listopada 54, Krakow, Poland; Department of Plant Biology and Biotechnology, University of Agriculture in Krakow, al. 29 Listopada 54, Krakow, Poland; Department of Biological, Ecological, and Earth Sciences, University of South Carolina Aiken, Aiken, SC, USA; Department of Biological, Ecological, and Earth Sciences, University of South Carolina Aiken, Aiken, SC, USA; Department of Plant Biology and Biotechnology, University of Agriculture in Krakow, al. 29 Listopada 54, Krakow, Poland

## Abstract

Transposable elements constitute a large portion of plant genomes and, due to their ability to change their genomic localization, they largely contribute to genome evolution and adaptability. Miniature inverted-repeat transposable elements (MITEs), due to their small size and localization near genes, seem to be a major source of potential functional variability. Effects imposed by MITEs on the expression of associated genes through redistributing *cis*-regulatory elements have been postulated, but our knowledge in this area still remains limited. We showed that MITEs in the carrot genome are enriched with binding sites for LHY/RVE transcription factors (TFs). Experimental validation using DcLHY-DAP-seq not only confirmed the enrichment of DcLHY binding sites within MITEs but also demonstrated that elements from the *DcTourist_15* family likely play a key role in redistributing these TF binding sites. We showed that insertional polymorphisms of *DcTourist_15* correspond with changes in the expression of associated genes, both in control conditions and in response to heat stress. In addition to placing individual genes under the control of DcLHY/RVE TFs, *DcTourist_15* copies were found in promoters of genes involved in sulfur metabolism and cysteine biosynthesis. The enrichment of rice MITEs in OsLHY binding sites suggests the phenomenon of MITE-driven rewiring of LHY/RVE regulation may be more widespread across the plant kingdom. Carrot MITEs, particularly those from the *DcTourist_15* family, drive evolution of the carrot genome, especially in the context of stress responsiveness, as they possibly fine-tune gene expression by redistributing binding sites for TFs from the LHY/RVE family.

## Introduction

The coding portion of the genome in plants is ~100–200 Mb. However, the genome size varies dramatically between genera and even species, ranging from the 119.1 Mb of *Arabidopsis* to 159 Gb of *Paris japonica*. Most genomes, including carrot, fall within the range of 400 Mbp–2 Gb [3]. It is known that polyploidy and transposable elements (TEs) are mainly responsible for these differences [4]. The proportion of repetitive sequences in the smallest genomes ranges from 17% in *Arabidopsis* [*5*], through intermediate levels in carrot (53%; [6]), up to extremely high amounts in species with large genomes, such as maize and wheat (~85%; [7]). TEs are so abundant due to their ability to proliferate within the host genome. Based on the mechanism of transposition, they are divided into two classes: Class I retrotransposons (RTs), which are mobilized through a copy-and-paste mechanism via an RNA intermediate, and Class II DNA transposons, which predominantly use a cut-and-paste transposition mechanism. Small, nonautonomous derivatives of DNA transposons carrying terminal inverted repeats (TIRs) are jointly referred to as miniature inverted-repeat transposable elements (MITEs). They are often AT-rich, enriched in the vicinity of genes, and can be mobilized by their autonomous relatives [8].

The frequent occurrence of MITEs close to coding regions, and their ability to change location within the genome, generates new variability [9]. MITE-derived variants often have a deleterious impact on the host genome, making it necessary for the host to strictly regulate their activity in order to maintain genome integrity. As rice is a model species that, unlike *Arabidopsis*, contains a large number of diverse MITEs, global studies on the impact of MITEs on the expression of nearby genes have been reported for this species. Among others, genome-wide analyses have shown that genes associated with MITEs exhibit significantly lower expression levels [10]. Like other TEs, MITEs can be epigenetically silenced through TE-derived 24-siRNA RNA-directed DNA methylation (RdDM), which in turn may have regulatory implications on nearby genes, explaining the lower expression of MITE-associated genes [9, 11]. However, MITEs can also influence the expression of nearby genes through other mechanisms, including methylation-dependent modifications of tissue-specific chromatin loops [12] and *cis*-regulation of circular RNA expression, which was shown to affect the expression of a gene responsible for ethylene production [13]. In rice, >300 protein-coding genes have coding sequences, polyadenylation sites, transcription start sites, and splicing sites that overlap with MITEs [14]. MITEs localized within the 3’-UTR can repress translation [15] and affect mRNA stability [16]. Additionally, insertions within gene sequences can lead to the generation of new splice variants [17] or contribute to the formation of *cis*-regulatory elements [18, 19].

It has been reported that TEs may provide *cis*-regulatory elements to the nearby genes, and the phenomenon has been documented for RTs that carry motifs used for their own regulation. Examples of such TEs include the *Arabidopsis* long terminal repeat (LTR)-RTs *ONSEN* and *Copia-35*, which contain heat-responsive elements within their LTRs. These elements can in turn confer heat responsiveness to neighboring genes, potentially leading to the formation of new *cis*-regulatory motifs or rewiring of preexisting transcriptional networks [20]. In common wheat, a substantial portion of subgenome-divergent transcription factor binding sites (TFBSs) originated from a differential expansion of specific LTR-RTs in the diploid progenitors, and they contribute to subgenome-divergent transcription [21]. Unlike RTs, MITE mobilization relies primarily on their TIRs, which are recognized by a compatible transposase encoded by a related autonomous element, while their internal regions do not generally contain features essential for their life cycle. However, previous reports point at the presence of several TF binding motifs in different MITE families of tomato, peach, and other *Prunus* genomes [19], as well as in *Brassica* species [18]. Although there is some evidence for the *cis*-regulatory role of MITEs in explaining changes in the expression of genes associated with MITEs [22], the importance of *cis*-regulatory elements in MITEs is still understudied, and mechanisms of evolution behind this phenomenon are not well described.

All living organisms have adapted their biological functions to the Earth’s circadian rhythm. Plants, due to their inability to move and change location in response to the time of day—and the associated fluctuations in temperature, light availability, humidity, and pest activity—not only synchronize their fundamental metabolic functions with the day–night cycle but also exhibit rhythmic responses to potentially adverse environmental conditions. The proper synchronization of these processes with the circadian rhythm is governed by the biological clock, which consists of the central oscillator as well as input and output pathways. The core genes of the central oscillator encode Myb-like TFs, including *CCA1 (CIRCADIAN CLOCK ASSOCIATED1)* and *LHY (LATE ELONGATED HYPOCOTYL)*, which are expressed in the morning and repress evening-expressed *PSEUDO-RESPONSE REGULATOR (PRR)* genes, such as *PRR1/TOC1 (TIMING OF CAB EXPRESSION1)*, *PRR5*, *PRR7*, and *PRR9*. In turn, TOC1 and other PRR proteins repress the expression of morning-expressed TFs, forming a feedback loop [23]. The Myb-like TF family also includes other REVEILLE (RVE) proteins, which can function as both activators [24] and repressors [25]. These proteins recognize similar DNA motifs and may compete for binding sites [26]. As key components of the circadian clock, LHY, CCA1, and other RVE proteins regulate processes such as growth, flowering, shade avoidance, and stress responses [27, 28]. Consequently, interactions among these proteins and their impact on gene expression are extensive and complex. Furthermore, recent analyses of knockout mutants of genes encoding RVE TFs have revealed that *CCA1* and *LHY* are epistatic to *RVE4*, *RVE6*, and *RVE8* in regulating the circadian clock and flowering time, while they interact additively to regulate growth. This suggests that these five Myb-like factors contribute differently to circadian clock regulation and growth control [29].

The carrot genome contains a large number of highly diverse MITEs [17]. They are also highly polymorphic, but the distribution of some copies can be associated with particular gene pools, i.e., wild, eastern, and western cultivated types [30]. Their proximity to genes and their genomic distribution patterns may suggest a potential regulatory role. Therefore, we aimed to determine whether carrot MITEs carry *cis*-regulatory elements and if they can affect the expression of adjacent genes. Here, we report on a significant enrichment of motifs bound by LHY/RVE proteins within carrot MITEs, a finding further confirmed experimentally through DAP-seq analysis, which demonstrated frequent colocalization of MITEs and DcLHY binding sites. Moreover, we observed a similar colocalization pattern between OsLHY DAP-seq peaks and MITEs in rice. However, unlike rice, we showed that in carrot, a single MITE family, *DcTourist_15*, likely plays a significant role in redistributing DcLHY binding sites within the genome. Additionally, we identified *DcTourist_15* copies capable of binding LHY/RVE TFs and affecting the expression of nearby genes in heat-stressed plants, in a manner corresponding to changes in the expression of these TFs.

## Results

### Transcription factor binding sites with AT-rich motifs are enriched in MITEs

We scored 10-nt-long k-mers across the carrot genome and within MITEs (Table S1). In total, 6606 k-mers (2.9%) were significantly enriched in MITEs. Of the k-mers enriched in MITEs, 699 k-mers showed significant similarity to known plant TFBSs (TomTom e-value <0.05, Table S3). For each k-mer significantly similar to a TFBS, we calculated the GC content. MITEs are AT-rich, which was also evident with respect to the k-mers enriched in MITEs. The average GC content of these k-mers was 12%, with 64% of k-mers containing only one G or C in their 10-nt-long sequence (Table S2). Next, we counted all occurrences in MITEs for each k-mer sequence, taking into account the classification of TEs into families. The resulting file was used to attribute k-mers occurring in MITEs to superfamilies (*Stowaway*, *hAT*, *Tourist*, *Mutator*) and families (Table S3). *Mutator* families carried the highest number of MITE-enriched k-mers (219), while each of the other superfamilies carried more than a hundred of such k-mers (112–127) (Fig. S1).

**Figure 1:**
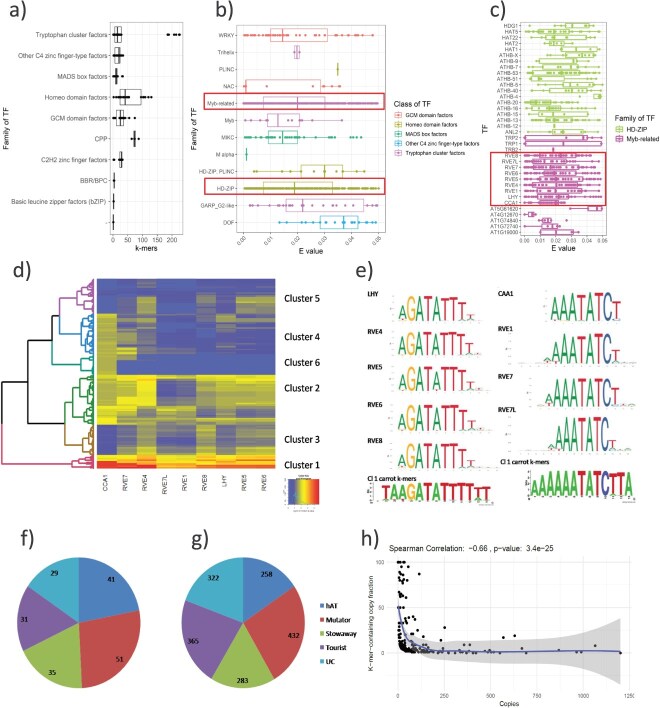
k-mers enriched in carrot MITEs potentially provide biding sites for TFs. Attribution of MITE-enriched k-mers to TF families (a), TomTom e-values showing similarity of k-mers enriched in MITEs to TFBSs at the family level (b) and to binding sites of Myb-related and HD-ZIP TFs (c). Heatmap of –log 10 TomTom e-values, representing similarity of k-mers enriched in MITEs to TFBSs (d). Logos of carrot k-mers from Cluster 1 (Cl1) and the corresponding conserved motifs recognized by Myb-like LHY/REV TFs from the JASPAR database (e). The number of MITE families (f) and copies (g) carrying k-mers potentially recognized by Myb-like LHY/REV TFs, and relationship between family copy numbers and numbers of copies containing such k-mers. On heatmap (d), e-value = 1 (−log10 = 0) was attributed to k-mers not associated with TFs.

We identified 143 TFs that could potentially recognize k-mers enriched in MITEs as their binding sites (Table S4). On average, a single TF could potentially bind to 37 k-mers enriched in MITEs (based on the TomTom e-value), but it varied significantly among TFs (Fig. 1a, Table S4). The largest number of MITE-enriched k-mers putatively providing TFBSs, ranging from 185 to 226, was identified for members of the family of Myb-related TFs comprising LHY/RVE proteins. Also, the most significant hits were attributed to motifs recognized by Myb-related and HD-ZIP families (Fig. 1b). The highest number of motifs matching TFBSs with e-value = 0 (adjusted TomTom *P*-value) was within Myb-related TFs, in particular the LHY/RVE proteins (Table S3, Fig. 1c), hence we focused our further analysis on that family. We determined the genomic positions of MITEs carrying k-mers most likely providing LHY/RVE TFBSs (Cluster 1 on Fig. 1d). A total of 1660 MITE copies from 187 families (36% of all MITE families) contained such k-mers (Fig. 1f and g). These k-mers shared a perfect core nine-nucleotide-long motif likely recognized by LHY/RVE TFs (Fig. 1e). Within each MITE superfamily, there were few low copy number families with most copies carrying k-mers grouped in Cluster 1, while families with higher copy numbers comprised <10% of copies with k-mers attributed to Cluster 1. It suggests that mutability of internal AT-rich segments of MITEs might be a phenomenon driving formation and redistribution of binding sites for TFs targeting AT-rich motifs, including LHY/RVE (Fig. 1h, Table S5).

### Expression of *LHY/RVE* genes in carrot is affected by abiotic stresses

**Figure 2:**
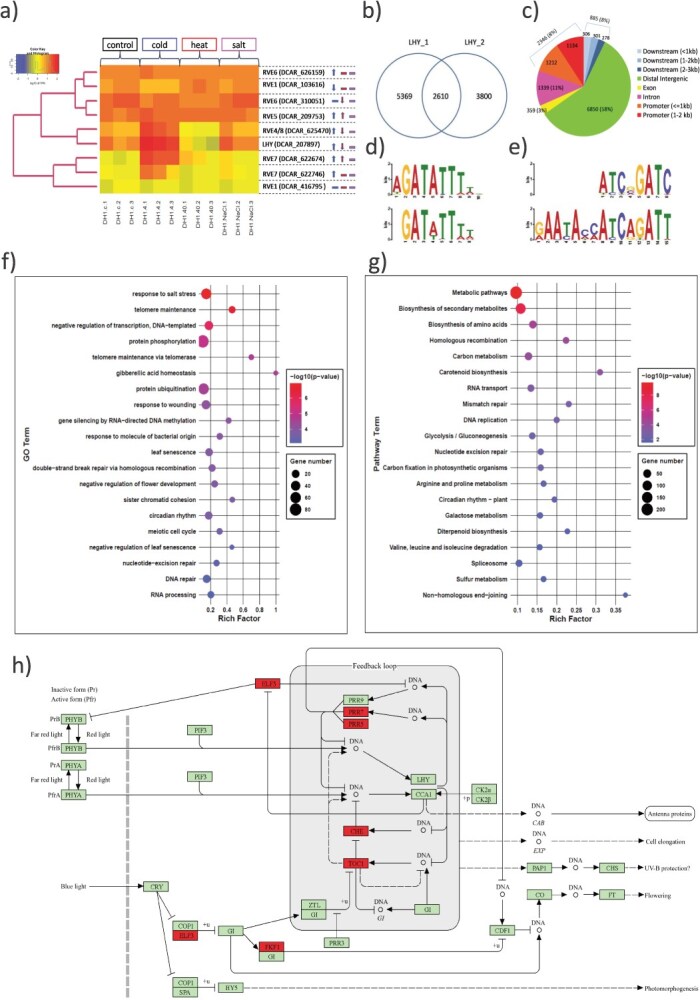
Expression and characteristics of the carrot LHY/RVE family. Expression of carrot *LHY/RVE* genes in cold-, heat-, and salt-stressed plants as compared to the control (a). Significant differences in gene expression are indicated by an arrow pointing up (upregulation) or down (downregulation), colored blue (cold), red (heat), and magenta (salt). Venn diagram of LHY binding sites called from two DAP-seq libraries (technical replicates) (b), positional annotation of LHY binding sites identified in both libraries with respect to genes (c), motifs present in carrot LHY-bound sites, similar to known TFBSs; LHY (d) and GATA20 (e). Top 20 GO terms assigned to biological function (BF) (f) and KEGG (g) terms for genes to which LHY binds in their promoter region. The circadian rhythm–plant pathway (ath04712) (h). Carrot genes with experimentally identified LHY binding sites in their promoters are shown in red.

To verify the postulated functional relationship between the putative TFBSs present within MITEs and the LHY/RVE TFs we first identified nine *LHY/RVE* genes in carrot and assigned them to their *Arabidopsis thaliana* homologs (Table 1). LHY/RVE TFs in plants are responsible for the regulation of the circadian clock. As their expression has also been shown to be affected by abiotic stresses [28], we investigated expression changes of *LHY/RVE* genes in leaves of young carrot plants subjected to 4 h of cold, heat, and salt stress. Principal component analysis (PCA) of normalized transcript expression revealed the distinctiveness of plants grown under cold stress, high temperature, and control conditions. In salt-stressed plants, no apparent changes in the expression of *LHY/RVE* genes were observed (Fig. S2). In contrast, cold and heat stress resulted in altered expression of most carrot *LHY/RVE* genes (Fig. 2a, Table S6). In cold-stressed plants, seven of nine *LHY/RVE* genes were differentially expressed and only one of them, *DcRVE1* (DCAR_416795), was downregulated. In heat-stressed plants, three genes were downregulated (*DcLHY;* DCAR_207897, *DcRVE6;* DCAR_310051 and *DcRVE4/8;* DCAR_625470), while two were upregulated (*DcRVE5*; DCAR_209753 and *DcRVE7;* DCAR_622674) (Table 1, Fig. 2a). Interestingly, *A. thaliana* homologs of all downregulated genes were characterized as activators while those upregulated were characterized as repressors of the downstream genes (Table 1).

**Table 1:** Carrot *LHY/RVE* gene family

Carrot *LHY/RVE*	TF ID (JASPAR)	*A. thaliana* homolog	Mode	ID UniProt	Reference
DCAR_207897	MA0972.1	*CCA1*	Repressor/activator	P92973	[31]/[32]
		*LHY*		F4HQG9	
DCAR_103616	MA1184.1	*RVE1*	Repressor/activator	F4KGY6	[33]/[34]/[35]
DCAR_416795	MA1184.1	*RVE1*	Repressor/activator	F4KGY6	[33]/[34]/[35]
DCAR_625470	MA1187.1 MA1182.1	*RVE4*	Activator	Q6R0G4	[24]
		*RVE8*		Q8RWU3	
DCAR_209753	MA1190.1	*RVE5*	Repressor	C0SVG5	[25]
DCAR_626159	MA1183.1	*RVE6*	Activator	Q8H0W3	[24]
DCAR_310051	MA1183.1	*RVE6*	Activator	Q8H0W3	[24]
DCAR_622746	MA1401.1	*RVE7*	Repressor	B3H5A8	[36]
		*RVE7L*	-	F4J2J6	-
DCAR_622674	MA1191.1	*RVE7*	Repressor	B3H5A8	[36]
		*RVE7L*	-	F4J2J6	-

### LHY/RVE regulate the expression of genes involved in response to abiotic stresses

We used DAP-seq to perform genome-wide identification of all possible DcLHY binding sites. Three libraries (two technical replications labeled LHY1 and LHY2, and the control input) were sequenced using Illumina NovaSeq in 150 PE mode (Tables S7 and S8). To ensure that the correct sequence was used for the *in vitro* synthesis of the protein encoded by *DcLHY*, its coding sequence was assembled from RNAseq data produced for leaves of 4-week-old carrot plants; grown under control, cold, heat, and salt stress; and compared to the genome annotation. The assembled coding sequence of LHY did not differ from that provided by the annotation (File S1).

Using DAP-seq, we identified 11 779 DcLHY binding sites, of which almost 20% (2346) were located in promoters of protein-coding genes (Fig. 2b and c, Table S9). DcLHY DAP-seq peaks sites were enriched in motifs similar to *Arabidopsis* LHY binding sites (Jaspar matrix MA1185.1), represented by the core sequence ‘GATWTTTT’ (MEME e-value = 1.6e-027, TomTom *P*-value =3.54e-09) with 99.9% of all identified sites contributing to the construction of the motif (11 766 of 11 779). If compared to the canonic *A. thaliana* motifs recognized by LHY/RVE TFs, DcLHY had no strong preference for the first nucleotide that is preferentially A in the case of Arabidopsis, and the fourth nucleotide (A/T) is less conserved (Fig. 2d). The second most enriched motif identified in DAP-seq regions was similar to the TFBS of GATA20 (MEME e-value = 4.30e-032, TomTom *P*-value = 3.25e-04) with much fewer sites (1169; 9.9%) contributing to the construction of the motif (Fig. 2e). It is consistent with the presence of an evening element (EE; AAATATCT) or GATA (HGATAR) motif within LHY/RVE binding sites in other plants [37] and demonstrates that DAP-seq efficiently and accurately identified DcLHY binding sites.

Gene ontology (GO) and Kyoto Encyclopedia of Genes and Genomes (KEGG) enrichment analysis for genes directly targeted by DcLHY (with binding sites in their promoters) indicated that it regulates the expression of genes involved in the circadian rhythm (GO:0007623; KEGG:ath04712), including its known targets such as *TOC1* (DCAR_518947) and other two-component response regulators (*PRR7*; DCAR_415535, *PRR5*; DCAR_416353), CHE (DCAR_209482), and *EARLY FLOWERING 3* (*ELF3*; DCAR_100582). In addition, carrot *FLAVIN-BINDING, KELCH REPEAT, F-BOX 1* (*FKF1*; DCAR_832785), which is not a direct target for LHY regulation in *Arabidopsis*, had DcLHY binding sites in its promoter (Fig. 2f and g, Fig. S6, Table S10, Table S11).

Besides the regulation of the circadian rhythm, DcLHY and other LHY/RVE TFs regulate responses to biotic and abiotic stresses. Three of 20 enriched GO biological processes were assigned to response to stress (response to salt stress KEGG: GO:0009651, response to wounding KEGG: GO:0009611, response to molecule of bacterial origin KEGG: GO:0002237). DcLHY binding sites were also present in genes from the carotenoid biosynthesis pathway (GO: KEGG: ath00906, Fig. S3), particularly those involved in carotenoid catabolism leading to abscisic acid biosynthesis (ABA), known to be involved in the regulation of the plant response to stress, and processes regulating gibberellic acid homeostasis (GO:0010336). Genes associated with the mitigation of negative effects of stress, such as DNA damage or abnormal telomere maintenance might also be controlled by DcLHY, as indicated by four KEGG pathways and five GO biological processes, i.e., double-strand break repair via homologous recombination (GO:0000724), nucleotide-excision repair (GO:0006289), DNA repair (GO:0006281), homologous recombination (KEG:ath03440), mismatch repair (KEGG:ath03430), nucleotide excision repair (KEGG:ath03420), nonhomologous end-joining (KEGG:ath03450), telomere maintenance (GO:0000723), and telomere maintenance via telomerase (GO:0007004). DcLHY may also regulate expression of other TFs, especially negative regulators of transcription (GO:0045892), related to negative regulation of flower development (GO:0009910) and leaf senescence (GO:0010150, GO:1900056), and other regulatory genes that are involved in gene silencing by RNA-directed DNA methylation (GO:0080188), RNA transport (KEGG:ath03013), splicing (KEGG:ath03040) and processing (GO:0006396), protein ubiquitination (GO:0016567), protein phosphorylation (GO:0016567), and karyokinesis (sister chromatid cohesion; GO:0007062, meiotic cell cycle; GO:0051321, DNA replication; KEGG:ath03030). These enriched functions are consistent with the enrichment of genes in corresponding cellular localizations and molecular function GO categories (Fig. S4a and b).

Next, we investigated the expression of genes from the 20 KEGG pathways and GO biological processes enriched under abiotic stresses. The highest number of differentially expressed genes (DEGs) was observed for the heat stress (10 901 DEGs; 31% of all annotated genes), followed by cold (2966; 8%) and salt (968; 3%) (Table S6, Fig. S5). Within 555 genes representing the top 20 enriched KEGG pathways and 412 genes representing the top 20 enriched GO biological processes, 50% (280) and 48% (199) responded to at least one of the applied abiotic stresses (Table S12). The highest fraction of DEGs likely regulated by DcLHY, from enriched pathways and biological processes, was also observed under the heat stress (Figs S6 and Fig. S7). However, some of the enriched pathways and processes contained a much higher fraction of stress-responsive genes, e.g., almost 60% of genes assigned to the circadian clock pathway were differentially expressed under both cold and heat stress. Stress-responsive DEGs attributed to carotenoid metabolism, sulfur metabolism, and amino acid metabolism were also abundant (Fig. S6). In contrast, genes involved in telomere maintenance and nonhomologous end-joining, associated with DcLHY binding sites, did not respond to the abiotic stresses.

### LHY/RVE binding sites overlap with MITEs in carrot and rice

In total, 1481 carrot MITEs (4% of all copies) overlapped with 1429 of 11 779 DcLHY binding sites (12%) identified with DAP-seq (Tables S13 and S14). To validate the result, we generated five sets of random MITE-like segments mimicking the actual genomic distribution of MITEs. The frequency of random MITE-like segments overlapping with DAP-seq peaks was much lower, ranging from 721 to 782 (2%) (Table S14).

**Figure 3:**
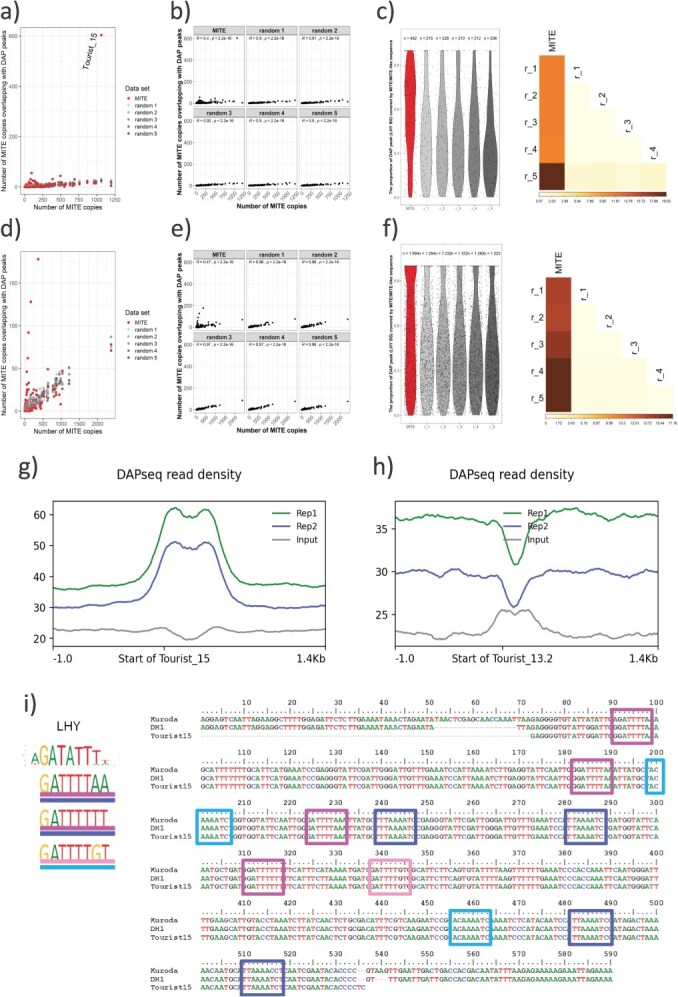
Co-occurrence of LHY binding sites and MITEs in carrot and rice. The number of MITEs per family and the number of random MITE-like sequences (r_1-r_5) overlapping LHY binding sites in carrot (a, b) and rice (d, e). Violin plots showing fractions of MITEs and random MITE-like sequences (r_1-r_5) overlapping LHY binding sites in carrot and rice, respectively, right panel, and -log10 BH-adjusted *P*-values indicating significance of differences in the mean lengths of MITEs and random MITE-like sequences (r_1-r_5) overlapping LHY binding sites in carrot and rice, respectively, left panel (c, f). Distribution of DAP-seq peaks along *DcTourist_15* (g) and *DcTourist_13.2* (h). Sequence of an exemplary copy of *DcTourist_15* in a promoter of *bHLH130* (DCAR_207114) (i)*.* Frames highlight motifs similar to the DcLHY binding site, the blue color corresponds to the reverse complement of the motif presented on the left.

Co-occurrence of MITE and MITE-like segments with DcLHY DAP-seq peaks corresponded with the MITE family copy number (Table S13). While no outliers were observed for all random MITE-like segments, copies from the *DcTourist_15* family much more frequently overlapped with DcLHY DAP-seq peaks (Fig. 3a and b), accounting for 57% of all copies from this family (Table S13). DAP-seq peaks covered a higher portion of ‘true’ MITEs, with a mean of 60%, as compared to the random MITE-like segments, with the mean ranging from 46% to 49% of the segment length (Fig. 3c and d, *P*-value <0.01; −log10 *P*-value >2). The differences were even more pronounced for the *DcTourist_15* family, where on average 77% of the element copies overlapped with DAP-seq peaks vs 48%–51% overlap for the simulated ones.

We performed a similar analysis in rice, using publicly available DAP-seq data [2]. The size of the rice genome is similar to that of carrot and it also contains many MITE families with a large number of copies [38]. We observed less evident enrichment of DAP-seq peaks in MITEs (5%; 1694 of 32 655 annotated copies), as compared to random MITE-like segments (4%, 1232–1292). However, the higher frequency of random MITE-like segments overlapping with DAP-seq peaks may be due to the fact that almost twice as many peaks were identified in rice than in carrot (19 905 vs 11 779) which, given the similar genome size, increased the chance for random overlaps (Tables S15 and S16). Nevertheless, colocalization of rice MITE copies and DAP-seq peaks was also observed for a few MITE families, but to a lesser extent than that for the *DcTourist_15* family in carrot (Fig. 3). This suggests that redistribution of TFBSs by MITEs may be a general mechanism rewiring plant regulatory networks.

### *DcTourist_15* is bound by DcLHY

Our results indicated that DcLHY binding sites in carrot were enriched in *DcTourist_15*. The genomic distribution of *DcTourist_15* copies was similar to the distribution of DAP-seq peaks, with higher density in gene-rich regions (Fig. S8). *DcTourist_15* is the second most abundant MITE family in carrot, with 1064 copies in the reference genome DH1, following the most numerous *DcTourist_13.2* family (Fig. S9a, Table S14). Thus, we sought to compare *DcTourist_15* to *DcTourist_13.2* with respect to their propensity to redistribute DcLHY binding sites. The two families differed sharply with respect to their association with DcLHY DAP-seq peaks, as only five copies of *DcTourist_13.2* overlapped with those peaks, vs 592 copies of *DcTourist_15* (Fig. S9b). *DcTourist_15* copies overlapping with DcLHY DAP-seq peaks were evenly distributed among older, more diverse copies, as well as the youngest ones, i.e., those exhibiting the smallest genetic distance (Fig. S9c).

**Figure 4:**
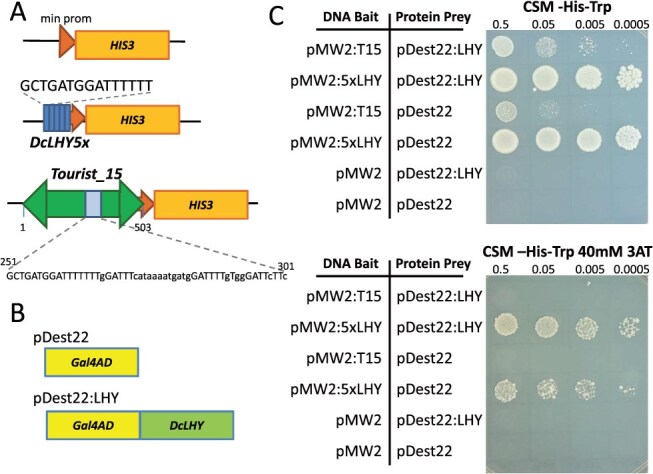
Analysis of DcLHY binding. Three ‘bait’ DNA constructs, pMW2 (top, control), pMW2:5xLHY (middle, with 5 DcLHY binding motifs), and pMW2:T15 (bottom, containing the *Tourist_15* element) (a) were tested for interaction with the activation domain alone (pDest22) or the activation domain DcLHY: fusion protein (pDest22:LHY), used as a ‘prey’ (b). Transformed yeast cells containing both ‘prey’ and ‘bait’ were plated on the selective medium CSM-His-Trp (c) with either 0 mM (top) or + 40 mM 3AT (bottom).

We used a yeast one-hybrid (Y1H) assay to test the ability of motifs present in the *DcTourist_15* sequence (bait) to interact with a DcLHY:activation domain protein (prey) expressed from the pDEST22:LHY plasmid (Fig. 4). In this assay, interaction between the bait DNA sequence and prey protein induces increased expression of *HIS3*, resulting in increased growth on plates lacking histidine. To account for the autoactivity of some bait constructs due to interaction with native yeast TFs, a competitive inhibitor of HIS3, 3-amino-1,2,4-triazole (3AT), is added to the media. Expression of the DcLHY:activation domain protein resulted in increased growth for yeast containing either the DcLHY motif (five copies) or the *Tourist_15* element as bait sequences. Though hard to compare because of different background levels of autoactivation, the reporter gene activation was slightly weaker for *Tourist_15*, consistent with it only containing one perfect DcLHY core motif sequence and multiple less conserved DcLHY motif variants.

The frequency of *DcTourist_15* copies inserted in promoters was higher (42%) than the average observed for all carrot MITEs (28%), all *Tourists* (30%), and *DcTourist_13.2* (34%) (Table S1, Table S17, Fig. S10). Polymerase chain reaction (PCR) amplification of products spanning 28 and 34 insertion sites of *DcTourist_15* and *DcTourist_13.2* within promoter regions revealed that these regions were highly diverse in cultivated carrots. Notably, 16 (57%) and 12 (35%) of the *DcTourist_15* and *DcTourist_13.2* insertions failed to produce clear amplification profiles in at least one of the seven carrot breeding lines. Among the informative sites, the rate of fixed insertions (i.e., those present in all seven carrot breeding lines) for *DcTourist_15* was unexpectedly high, reaching 58%. In contrast, the *DcTourist_13.2* family had a lower rate of fixed insertions (45%). In both families, fixed and nonfixed insertions were randomly distributed on the neighbor-joining (NJ) tree (Fig. S11). This might suggest that either a fraction of *DcTourist_15* insertions have been under positive selection, or they have been more effectively purged due to their deleterious effects. As mentioned above, on average 77% of a *DcTourist_15* copy overlapped with DcLHY DAP-seq peaks and 18% of those copies were fully covered by a DAP-seq peak (Table S13). This demonstrates clear enrichment of reads covering *DcTourist_15* copies, as compared to their flanking regions while no such enrichment was observed for *DcTourist_13.2* (Fig. 3g and h). *DcTourist_15* copies carry motifs similar to *Arabidopsis* LHY binding sites, as well as motifs that are less similar to the canonical *Arabidopsis* sites, often carrying T at position four, which corresponds to A at position five in *Arabidopsis* (Figs 1e, 3i, and 4a). This may explain why *DcTourist_15* was not primarily identified in our k-mer-based analysis, which relied on the identification of the canonical *Arabidopsis* sites.

### *DcTourist_15* copies may redistribute LHY/RVE binding sites and rewire regulatory networks

Carrot genes with DAP-seq peaks and *DcTourist_15* insertions in their promoters present in DH1 were significantly enriched in GO terms or KEGG pathways, especially those related to the sulfur and cysteine metabolism, i.e., KEGG:ath00920, KEGG:ath00270 GO: GO:0019344, GO:0006535, and GO:GO:0004124 (Figs S12 and S13; Tables S18 and S19). All genes bound by DcLHY in the promoter region were also enriched in the sulfur metabolism category. Interestingly, six of the seven genes assigned to this category and involved in all steps of conversion of sulfate into L-cysteine contained insertions of *DcTourist_15* copies in their promoters (Tables S10, S11, S18, and  S19). Genes associated with *DcTourist_15* copies were involved in the L-cysteine conversion from/to L-serine and O-phospho-L-homoserine conversion into L-cystathione, a precursor of L-cysteine and L-methionine (Fig. S14). For all these genes, *DcTourist_15* copies overlapped with DcLHY DAP-seq peaks (Fig. S15).

As carrot is an allogamous species characterized by strong inbreeding depression, breeding lines are often characterized by relatively high levels of residual heterozygosity [39, 40]. Therefore, using 28 previously published DcS-ILP markers that identify intronic insertional polymorphisms of carrot *Stowaway* MITEs [1], we examined the degree of homozygosity of nine carrot breeding lines. 493B and Nh2168B were the most homozygous, as indicated by the lowest percentage of polymorphic loci and Ho (0.002 and 0.105; Table S20; Fig. S16). Importantly, PCoA revealed clear genetic distinctiveness of those lines and the reference line DH1 (Fig. S16). Therefore, to verify the effect of *DcTourist_15* copy presence/absence on the expression of adjacent genes, we used DH1 in combination with 493B and Nh2168B. We investigated the effect of heat stress on the expression of genes potentially controlled by LHY/RVE TFs through TBFSs provided by copies of *DcTourist_15*.

Firstly, using single nucleotide polymorphisms (SNPs) from the RNAseq data we confirmed high levels of homozygosity of the two selected lines (Figs S17 and S18; Table S21). Subsequently, MITE insertions were identified in each breeding line using whole-genome sequencing (WGS). A total of 5417 and 4198 high-confidence, homozygous reference MITE copies were identified in 493B and Nh2168B, respectively.

**Figure 5:**
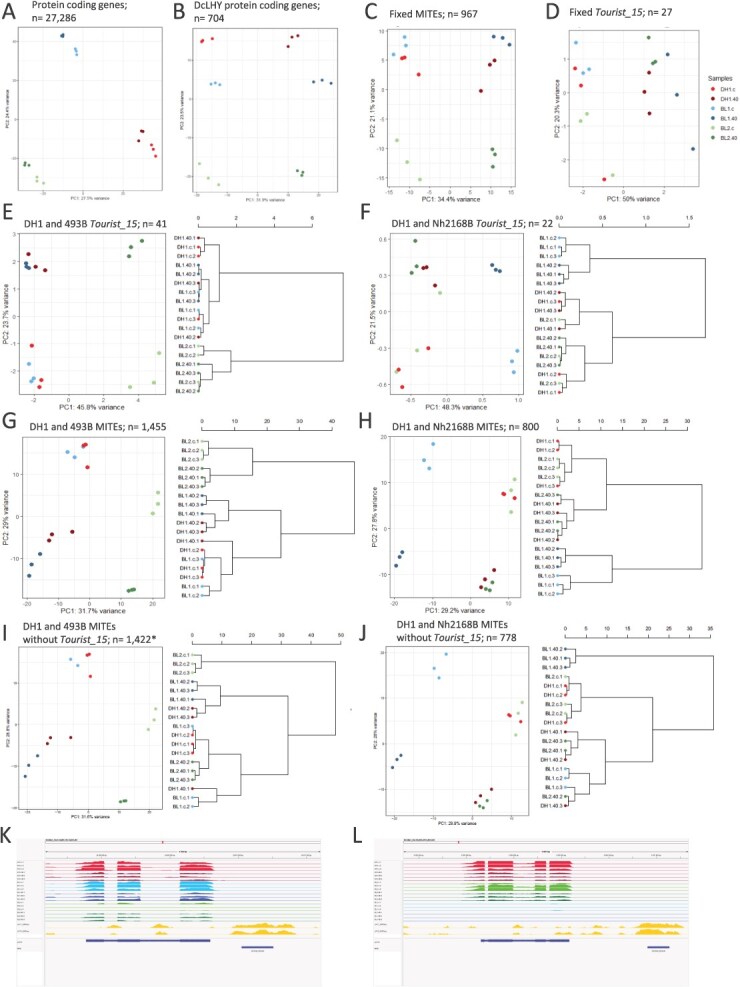
*DcTourist_15* copies may affect expression of nearby genes. PCA on normalized expression of carrot protein-coding genes in DH1, 493B, and Nh2168B grown under heat stress and control conditions; all genes (a), genes associated with DcLHY DAP-seq peaks (b), genes associated with fixed MITE copies (c), and genes associated with fixed *DcTourist_15* copies (d). PCA on normalized expression of carrot protein-coding genes in DH1, 493B, and Nh2168B grown under heat stress and control conditions along with a dendrogram representing hierarchical clustering with respect to PC1 of genes associated with *DcTourist_15* copies (e, f), all MITE copies (g, h), and all MITE copies but *DcTourist_15* (i, j) present in DH1 and 493B but absent in NH2168B, or present in DH1 and NH2168B but absent in 493B. * Eight genes with *DcTourist_15* insertions present in DH1 and 493B are retained due to the presence of other MITEs that meet the filtering criteria. Examples of genes with expression patterns altered by the presence of *DcTourist_15* copies in their promoter regions; DCAR_101531 (k) with a copy present in DH1 and 493B while absent in NH2168B; and DCAR_727564 (l) with a copy present in DH1 and NH2168B while absent in 493B. Both genes show higher expression when the *DcTourist_15* copy is present but also downregulation in response to heat, as expected from the general pattern of LHY/RVE expression. The tracks show expression levels (genome coverage by RNAseq reads in three biological replicates) in DH1, 493B (BL1) and Nh2168B (BL2), respectively, in control (pale) and heat (dark) and coverage by DAPseq reads (two technical replicates).

The global gene expression was genotype-dependent, and no apparent effect of heat stress was observed (Fig. 5a). In contrast, the expression of genes associated with DcLHY DAP-seq peaks was considerably more affected by heat (Fig. 5b).

The expression of genes associated with MITE, *DcTourist*, and *DcTourist_15* copies in the DH1 reference genome was also globally affected by heat stress (Fig. S19a-c), which was even more pronounced when only the genes associated with MITE copies fixed in the three lines were considered (Fig. 5d). For genes associated with those shared insertions, the percentage of variance explained by the reaction to heat stress increased and the most pronounced increase from 28% to 50% was observed for *DcTourist_15*, along with a decrease of the genotype effect on grouping (Fig. 5d, Table S22). Next, we investigated effects of nonfixed MITE insertions, i.e., those present in DH1 and 493B while absent in NH2168B, or in DH1 and NH2168B while absent in 493B, on the expression of adjacent genes. The lines carrying gene variants associated with the presence of MITE copies grouped together, while the line with empty sites was always separate. This suggests alteration of gene expression profiles in plants differing with respect to the MITE copy status (Fig. 5g–h). It was even more clear for genes associated with *DcTourist_15* copies that were likely under control of stress-responsive LHY/REV TFs, where the percentage of variance explained by PC1 (separating lines with insertions from the line with empty sites) reached 46% and 48%, for DH1/493B vs NH2168B and DH1/NH2168B vs 493B, respectively (Fig. 5e–f, Tables S23 and S24). In all cases, heat stress globally affected the expression of the genes in question. Importantly, when PCA was performed on all polymorphic MITEs but *DcTourist_15*, the clear distinction with respect to the presence or absence of MITE copies along PC1 could no longer be observed (Fig. 5i and j). It suggests that *DcTourist_15* copies provide *cis*-regulatory elements modifying the expression of associated genes.

We identified the top 10 genes associated with *DcTourist_15* insertional polymorphisms that mostly contributed to PC1 (Fig. S20). Changes in the expression of these genes did not reveal a common trend. We observed distinct expression patterns, including genes with higher expression associated with the presence of a *DcTourist_15* copy in the upstream regulatory region and downregulated by heat stress (Fig. 4k and l; DH1 BL1 - DCAR_101531; DH1 BL2 - DCAR_727564); downregulated genes associated with upstream *DcTourist_15* copies showing no reaction to heat stress (Fig. S21; DH1 BL1 - DCAR_521955); genes not expressed in genotypes lacking the insertion, but upregulated in response to high temperature in lines carrying a downstream *DcTourist_15* copy (Fig. S22; DH1 BL1 - DCAR_518578); and genes with downstream insertions that were not expressed when the associated *DcTourist_15* copy was present while showing low expression levels when it was absent (Fig. S23; DCAR_520595). However, regardless of the reaction to heat stress, the contrasting patterns were consistently maintained among the genes associated with the presence or absence of a *DcTourist_15* copy. Furthermore, in all cases, *DcTourist_15* copies exhibited high DAP-seq read coverage in both technical replicates while it was not observed in surrounding regions containing other MITE copies (Figs S21 and S22). Most genes associated with *DcTourist_15* copies could be functionally annotated but we have not found any significant enrichment with respect to metabolic pathways or biological processes, indicating that at least a fraction of the MITE/gene associations, especially those *DcTourist_15* copies that showed insertional polymorphism, were likely not providing any advantage to the plants’ reaction to stress.

## Discussion

The mobilization of TEs generates novel structural variants, including those located in the vicinity of coding regions and possibly having an impact on the expression of neighboring genes. As a result, in the face of drastic environmental changes or the colonization of new ecological niches, it is transposition—rather than incremental accumulation of random mutations—that becomes the driving force of plant evolution [41]. As MITEs are commonly located in coding regions of the genome, they are potentially an important source of new genetic variability [19]. However, most of these insertions are neutral or deleterious. In a scenario where a MITE family capable of reprogramming gene expression by providing *cis*-regulatory elements is actively transposing, most new copies associated with genes would be targets of purifying selection, unless they provide an adaptive advantage. In the latter rare cases the MITE/gene association would be positively selected for. This implies that two variants of such associations could be observed, (i) a well-established association providing advantage to the host and the MITE copy presence likely fixed in the genome, and (ii) a ‘spurious’ association resulting from a recent transposition event, resulting in altered gene expression that likely does not provide any adaptive advantage and shows MITE insertional polymorphism.

Although fine-tuning of gene expression by TEs is well documented, only a few MITE families have been shown to contain TFBSs. Such reports were based on *in silico* analyses [18, 19] or studies in which a *cis*-regulatory role was postulated from the MITE/phenotype correlation and the presence of motifs recognized by more than one TF [22]. Only a few MITE-embedded TFBSs were validated by an *in vitro* assay [18].

Here, using an *in silico* approach previously successfully applied for the identification of TFBSs redistributed by MITE in *Prunus* species [19], we demonstrated that LHY/RVE TFBSs are enriched in carrot MITEs. We also confirmed that the experimentally identified binding sites of DcLHY and OsLHY, in carrot and rice, respectively, overlapped with MITEs more frequently than with similarly positioned random DNA segments, as revealed by DAP-seq. Additionally, in the case of MITEs, the DAP-seq peak more frequently spanned entire elements. We were also able to experimentally confirm the interaction between DcLHY and the *DcTourist 15* MITE sequence using a Y1H assay. Our *in silico* analysis of k-mers enriched in carrot MITEs showed that hundreds of motifs exhibited similarity to the canonical LHY/RVE binding site. This finding aligns with the fact that LHY and RVE proteins share the same consensus sequence, SHAQK(Y/F)F, in their DNA binding Myb-like domain [26]. Because it is easier to reproduce multiple low-affinity binding sites than to maintain a single high-affinity site [42] it is possible that the enrichment of LHY binding sites in MITE copies representing different families is a result of random variants present in generally AT-rich sequences, leading to an increased frequency of the motif that can be recognized by TFs binding to AT-rich sites, e.g., LHY/RVE. Indeed, the siRNA-directed TE methylation followed by deamination of methylated cytosines to thymines increases C to T conversion rates in TEs [43]. Additionally, mutation rates may raise under unfavorable conditions and increased mutation frequencies was attributed to TEs [44].

Recently, Borredá *et al*. [45] utilized DAP-seq data for 529 *Arabidopsis* TFs to investigate whether and which TFs regulate the expression of genes encoded by TEs. Their results demonstrate that RTs, particularly those from the *Ty1/Copia*and *Ty3/Gypsy* families, tend to carry TFBSs upstream of transcription start sites (TSSs), whereas TFBSs in DNA transposons are often located downstream of TSSs. Furthermore, different TE families, as well as genes within TE copies, have evolved unique transcriptional regulation mechanisms through the presence of specific *cis*-regulatory elements. It is therefore possible that the TFBSs identified in MITEs were originally present in their ancestral autonomous copies and played a role in regulating the expression of their transposases. A newly identified indel-containing MITE associated with a maize *PIP* gene was shown to alter the expression of that gene, most likely due to presence of *cis*-regulatory elements [22]. Interestingly, we searched for possible TFBSs in the *PIP*-associated MITE and revealed that, among others, a putative RVE4 binding site was present (data not reported), which further supports the results we reported for carrot and rice. It jointly suggests that redistribution of LHY/RVE TFBSs by MITEs may be a common mechanism for rewiring or fine-tuning plant regulatory networks.

What seems to be unique to carrot is that a single MITE family, *DcTourist_15*, appears to be actively spreading DcLHY binding sites along the host genome. For this family, positions of nearly 60% of copies overlapped with DcLHY binding sites in the carrot reference genome DH1. We observed that *DcTourist_15* copies capable of binding DcLHY were scattered along the genetic distance tree, possibly pointing at the ancestral origin of the LHY/RVE TFBS. Therefore, it is likely that copies nonoverlapping with DAP-seq peaks lost their ability to bind DcLHY due to the gradual accumulation of mutations.

TFs constituting the LHY/RVE family can recognize similar motifs and, therefore, may compete for the same TFBSs in the genome. Since the LHY/RVE proteins include both activators [24] and repressors [25], their binding is usually tightly regulated. For example in *Arabidopsis*, under normal conditions, CCA1 and LHY acting as repressors are bound to the EE in the promoter of *DREB1A*. However, under cold conditions, positive regulators of expression, RVE4 and RVE8, are preferably bound. This is coupled with modifications of RVE4/8 leading to their efficient transport from the cytoplasm to the nucleus while at the same time CCA1 and LHY are degraded [46]. With respect to fine-tuning gene expression by MITEs carrying LHY/RVE TFBSs, such precise control mechanisms, as well as a strictly defined direction and magnitude of gene expression changes, should not be expected. Instead, a more random response might be anticipated, reflecting altered expression of genes randomly associated with recent insertions of copies carrying LHY/RVE TBFS. We assumed that the direction and magnitude of gene expression changes would vary, thus, we decided to use PCA to reveal the global effects of MITEs. Our results pointed to the stress responsiveness of genes associated with MITE insertions, especially *DcTourists_15*, although there was no clear shift toward up- or downregulation. Importantly, PCA clustering of carrot plants based on the expression of all genes potentially regulated by LHY/RVE revealed grouping of plants from different lines and clear impact of heat stress. In contrast, PCA clustering with respect to the expression of genes associated specifically with *DcTourist_15* insertions primarily reflected the reaction to stress in the insertion presence/absence context, with a marginal effect of the plants’ lines of origin. This suggests that it is the presence of a *DcTourist_15* copy that places genes under the control of LHY/RVE TFs, leading to similar expression levels under control conditions and comparable shifts in expression in response to stress in plants carrying the insertion. We showed that the altered expression of carrot genes associated with *DcTourist_15* insertions was not only induced by stress but was also observed in the absence of stress, as confirmed by the clear separation along PC1 of plants with respect to their insertion presence/absence status, indicating that gene expression depends on *DcTourist_15*. Our results are consistent with those of global analysis of TE-associated expression Quantitative Trait Locus (eQTLs) in rice showing that TE insertions shared among populations provided incremental changes in expression of nearby genes while the most pronounced effects on the expression were associated with rare variants, likely due to their deleterious effects [47]. Those TE insertions were associated with genes related to adaptability and domestication, despite having relatively little impact on gene expression. A low number of fixed *DcTourist_15* insertions and their usually small effect on the expression of adjacent genes corroborates previous reports in carrot and other plants on high levels of MITE insertional polymorphisms, large numbers of private insertions, even among closely related accessions, and low frequency of fixed insertions, which were mostly attributed to older MITE families [30, 48].

Localization of DAP-seq peaks across the genome in the carrot DH1 line revealed that genes involved in sulfur metabolism were possibly regulated by DcLHY (Fig. S15). Sulfur metabolism has not been previously reported as being under control of LHY/RVE TFs. Interestingly, six out of the seven genes assigned to the sulfur metabolism pathway also carried upstream *DcTourist_15* insertions. Thus, it is *DcTourist_15* copies that likely provided the LHY/RVE TFBSs. However, as discussed above, no apparent changes in the expression of those genes in heat-stressed DH1 plants were observed. It remains to be investigated if these *DcTourist_15*/gene associations provide any adaptive advantage, e.g., related to the core circadian clock regulatory function of LHY/RVE TFs.

## Conclusion

These results provide new insights into how carrot MITEs, particularly those from the *DcTourist_15* family, contribute to carrot genomic diversity and show that they can potentially drive adaptation to environmental stressors. *DcTourist_15* may help fine-tune gene expression through providing *cis*-regulatory elements, namely binding sites for TFs from the LHY/RVE family. Insertion of *DcTourist_15* into a regulatory region can alter the expression of the insertion-associated gene, rendering it stress-responsive. However, the MITE/gene associations are occurring at random, as a consequence of mobilization, and if disadvantageous, they are likely purged. Thus, most of the polymorphic *DcTourist_15* insertions affecting gene expression are likely recent and they possibly do not provide any obvious fitness benefit to the host. However, the fact that in the carrot DH1 line most genes involved in sulfur metabolism, regulated by *DcLHY*, were associated with *DcTourist_15* insertions suggests that these MITEs may drive the emergence of a novel regulatory network controlled by LHY/RVE TFs, highlighting their potential role in the evolvability of the carrot genome.

## Materials and methods

### Plant materials

DH1, a double-haploid line used to assemble of the carrot reference genome (GenBank GCA_001625215.2; [6]) and nine carrot breeding lines provided by P.W. Simon (USDA-ARS, Vegetable Crops Research Unit, University of Wisconsin-Madison, USA) were used in this study (Table S25). Seeds were sown in multi pots. Plants were grown in a growth chamber under a 16-hour light/8-hour dark photoperiod at 20°C. To assess homozygosity of the breeding lines, genomic DNA was extracted from 20 plants per line using the CTAB method and the plants were genotyped with 28 *DcS*-ILP (*Daucus carota Stowaway*-like Intron Length Polymorphism) markers developed by [1] (Table S26). Homozygosity of 493B and Nh2168B was evaluated in more detail with BAM files generated from RNA-seq (see below). Duplicates were flagged using the PICARD MarkDuplicates tool (http://picard.sourceforge.net/), and SNPs were called using the Genome Analysis Toolkit (GATK; [49] following the GATK best practices workflow (https://gatk.broadinstitute.org/hc/en-us/articles/360035531192-RNAseq-short-variant-discovery-SNPs-Indels). Long runs of homozygosity were detected using the R package ‘detectRUNS’ [50], with parameters set to: windowSize = 10, threshold = 0.05, minSNP = 20, ROHet = FALSE, maxOppWindow = 1, maxMissWindow = 1, maxGap = 10^6, minLengthBps = 250 000, minDensity = 1/10^3. Genetic uniformity within lines was further assessed by estimating nucleotide diversity (PI) with VCFtools v.0.1.16 [51] software (−window-pi 50 000 -window-pi-step 10 000) and conducting PCA with PLINK v1.90b7.1 [52], followed by visualization using the R packages ‘graphics’ (R Core Team, 2022) and ‘ggplot’ [53].

### Stress treatments

To investigate the early response of carrot plants to abiotic stresses (heat, cold, and salinity), 4-week-old plants (2–4 true leaves stage) of DH1, 493B, and Nh2168B were subjected to stress treatments. The following conditions were applied: salinity stress – plants were flooded with 150 mM NaCl solution for 15 min. The solution was then drained, and plants were left under standard growth conditions; cold stress – plants were exposed to 4°C; heat stress – plants were exposed to 40°C at 60% relative humidity. All stress treatments were applied between 9 and 11 a.m. In Experiment 1, three bulks of leaf samples comprising 10 stress-treated DH1 plants were collected after 4 h of treatment while samples from control plants were collected at 1 p.m. In Experiment 2, leaf samples were collected from three heat-stressed and three control plants from three lines (DH1, 493B, and Nh2168B). All samples were immediately frozen in liquid nitrogen and stored at −80°C until further analysis. Total RNA was extracted using the Direct-zol™ RNA MiniPrep Plus kit with TRI Reagent (Zymo Research, Irvine, CA, USA).

### Analysis of carrot transcriptomes in response to abiotic stresses

A total of 30 RNA samples were prepared: 12 DH1 bulk leaf samples from plants grown under heat, cold, and salt stress, and in control conditions (each in three biological replicates), and 18 samples extracted from leaves of individual DH1, 493B, and Nh2168B plants grown under heat and control conditions. Samples with RNA integrity number (RIN) >7, as assessed by Bioanalyzer (Agilent Technologies), were used for library preparation and sequencing on the Illumina HiSeq4000 platform in 150-bp paired-end mode (Illumina, San Diego, CA, USA) at Novogene. RNA-Seq data have been deposited in the NCBI Sequence Read Archive (SRA) under BioProject PRJNA1234542 (Table S27). Impact of TE copies on the expression of nearby genes was assessed by performing PCA on normalized expression data of selected genes, utilizing R packages DESeq2 [54], ‘ggplot2’ [55], and ‘ggfortify’ [56]. Hierarchical clustering was done in R package ‘stas’ [57], based on the Euclidian distance calculated on PC1. Gene expression analysis and identification of DEGs were performed as described by [58], using the DH1 v3.0 carrot reference genome (GenBank assembly accession number GCA_001625215.2) and the annotation available at the Carrotomics website (https://www.carrotomics.org/bio_data/291891). The functional annotation was carried out using Trinotate [59].

### Genome-wide identification of MITEs in 493B and Nh2168B

Positions of MITE copies in the reference genome of DH1 were reported previously [17]. To identify MITE insertion sites in the two breeding lines (493B and Nh2168B), bulk leaf samples were collected from 20 plants per line. The plant material was immediately frozen in liquid nitrogen and stored at −80°C (Table S25). DNA extraction and WGS were performed by Novogene on the Illumina platform using 150 PE mode. MITE insertions were identified in WGS reads (Table S28) using RelocaTE2 with default parameters [60], v3.0 DH1 carrot genome as a reference (GenBank assembly accession number: GCA_001625215.2; [6]), and the carrot MITE library [17]. Insertion sites found in 493B and Nh2168B were combined and summarized using a custom R script (SFile2).

### Identification and annotation of k-mers enriched in MITE sequences

To identify k-mers enriched in MITEs, we developed and utilized k-mer_counter pipeline (available at https://github.com/Aviatore/kmer_counter.git) following the methodology described by [19]. Given that the size distribution of plant TF binding motifs shows a major peak between eight and 10 nucleotides [19], we first identified 10-nt k-mers in the carrot reference genome assembly DH1 v3.0 (GenBank GCA_001625215.2; [6]) using Jellyfish 2.2.10 [61]. Subsequently, we counted k-mer occurrences along each chromosome in two groups: within MITEs and in non-MITE regions, using kmer_counter.py. Values obtained for each chromosome were aggregated using tables_merger.py, and the merged data were used to identify k-mers enriched in MITE sequences using stats.py. This script calculated the abundance of each k-mer in all MITE and non-MITE sequences and compared the observed frequency of k-mers in MITE and non-MITE sequences with their theoretical abundances using Fisher’s exact test. The theoretical values were calculated by multiplying the total abundance of a given k-mer by the proportion of MITE and non-MITE sequences in which it occurred. Statistical significance was assessed using Bonferroni adjusted *P*-values calculated in R [57]. From the pool of k-mers with *P*-values <0.05, those with frequencies in MITEs at least 20-fold higher than the genome-wide were selected. The k-mers enriched in MITEs were compared to TFBS motifs deposited in the JASPAR_CORE_2016_plants database [62] using TomTom, a component of the Sequence Motif Analysis Toolkit (MEME; [63]). K-mers with e-value <0.01 were selected and annotated using data from the UniProt database [57, 64].

### Genome-wide identification of LHY binding sites

DNA affinity purification sequencing with DNA amplification (ampDAP-seq; [65]) was used to evaluate genome-wide localization of all potential LHY TF binding sites. DNA was extracted from the leaves of a carrot plant of the DH1 (double haploid) line, which was previously used for the reference genome assembly [6]. The DNA extraction was done using Oxford Nanopore protocol for high molecular weight gDNA from *Arabidopsis* (https://nanoporetech.com/document/extraction-method/Arabidopsis-leaf-dna). The coding sequence of *DcLHY*, used for the *ex vivo* synthesis of the DcLHY protein, was obtained from our RNAseq data (BioProject PRJNA1234542, described above), assembled with Trinity v2.14.0 [66] using –min_contig_length 100 (SFile1) and compared with the cds from the DH1 reference genome annotation (https://www.carrotomics.org/bio_data/291891; [6]). The DAPseq experiment was conducted by Profacgen (https://www.profacgen.com/). Three libraries (two technical replicates and the control input) were sequenced using the Illumina NovaSeq platform in 150 PE mode. Since a doubled haploid line was used for the study, the performed technical replicates can be considered as biological replicates. Clean reads were aligned to the carrot reference genome [6] using Bowtie 2 v.2.3.5.1 [67], and peaks were defined using MACS v.2 software using q = 0.05 [68]. Motifs enriched in DAP-seq peaks were identified using MEME Suite v4.11.4 [69]. Carrot MITE copies and DAP-seq peaks were colocalized with genomic features, such as promoters (defined as 2-kb-long regions upstream of transcription start sites), exons, introns, and regions 2 kb downstream of genes, using BEDTools v2.27.1 [70] with the –D parameter to report distances to the nearest feature, as described by [30]. Pathway and GO enrichment analyses were performed using KOBAS 3.0.3 [71] with the KEGG PATHWAY database [72] and the GO database [73]. The *A. thaliana* data were used as a reference. Input data comprised genes with DcLHY binding sites in their promoters, extracted using gffread v.0.12.7 [74] from the DH1 carrot reference genome [6]. Enriched pathways and GO terms were identified using Fisher’s exact test [75] and the hypergeometric test [76], with Benjamini–Hochberg false discovery rate (FDR) correction [77] applied to control for multiple comparisons. A significance threshold of *P* < 0.05 was used, and the 20 most significant terms were visualized using the ‘ggplot2’ package in R [55]. Distribution of genes, LHY binding sites and MITEs was plotted using Circos [78].

To verify the enrichment in LHY binding sites within MITEs, we created a random set of chromosomal positions equal to the number of MITEs identified on each chromosome. These numbers were considered as the start points of MITE insertions while their ends were determined by adding the length of the MITE to its start coordinate. Subsequently, the MITE copy used to generate the final coordinates was applied to assign these genomic segments to the respective MITE families. The coordinates of MITEs and random MITE-like sequences overlapped with DAP-seq peak coordinates. The generation of random genomic segments, summary statistics and visualizations, including the number of MITEs overlapping DAP-seq peaks, the fraction of MITE sequence overlapping DAPseq peaks, Shapiro–Wilk tests for data distribution, and pairwise Wilcox tests comparing fractions among MITEs and random MITE-like sequences, were performed using a custom R script (SFile3). The same strategy was used for rice, using DAP-seq data for LHY binding sites published by [2], the rice reference genome (RAP), and MITE annotations obtained from the same pipeline we previously used to annotate carrot MITEs [17].

### Yeast one-hybrid

Y1H assay was used to analyze the interaction between DcLHY and the *Tourist_15* copy localized on Chr8:13638552–13 639 014, for which two DAP-seq peaks were detected. This element contained one full copy of the DcLHY binding motif (GCTGATGGATTTTTT), along with several shorter variants within its sequence [capitalized in Supplementary File S1 (File S1)].

As ‘bait’ controls, we used five tandem repeats of the DcLHY binding motif (GCTGATGGATTTTTT) and the empty pMW2 plasmid [a gift from M. Walhout (Addgene plasmid # 13349; http://n2t.net/addgene:13349; RRID:Addgene_13 349)] [79]. The ‘bait’ DNA fragments were cloned into the *Mlu*I site of pMW2 using the NEBuilder HiFi DNA Assembly Master Mix (NEB) and subsequently digested with *Xho*I prior to integration into the YM4271 yeast genome. The DcLHY CDS (SFile1) was cloned into pDONR Zeo and transferred into the destination vector pDEST22 using BP and LR Clonase reactions (Invitrogen). Next, the control pDEST22 or pDEST22:LHY ‘prey’ constructs were transformed into the YM4271 yeast strains containing the genomic pMW2 reporter constructs using the LiAc method, and transformants were selected on CSM − Trp. Fresh liquid cultures of these transformants were diluted to 0.5 OD_600_ and 10 μl of a dilution series was spotted on CSM-His-TRP with varying levels of 3-AT (to reduce the background growth) and incubated at 30°C for 3–4 days.

### Analysis of *DcTourist_15* and *DcTourist_13.2*

DNA sequences of each copy belonging to *DcTourist_15* and *DcTourist_13.2* families were aligned using MAFFT v.7.453, with –auto –reorder parameters [80]. Kimura 2-parameter model [81] was used to calculate distance in MEGA-X [82], with pairwise deletion. A phylogenetic tree was constructed in MEGA-X [82] using the NJ method [83]. Bootstrap analysis [84] was conducted using 500 replicates.

## Supplementary Material

Web_Material_uhaf360

## Data Availability

RNA-seq and WGS-seq data have been deposited in the NCBI Sequence Read Archive (SRA) under BioProject PRJNA1234542 (Tables S27 and Table S28).
