# Supplementary material for: Cis-regulatory effects of carrot miniature inverted-repeat transposable elements on the expression of genes controlled by LHY/RVE transcription factors
Source: Hortic Res. 2026 Jan 2;13(4):uhaf360. doi: 10.1093/hr/uhaf360 (PMC13102513; doi:10.1093/hr/uhaf360)
Supplement: Web_Material_uhaf360 [file web_material_uhaf360.zip › SFig_MITE_LHY_HR.docx]

**SFigure 1.** Number of MITE families with the highest share of MITE-enriched k-mers.


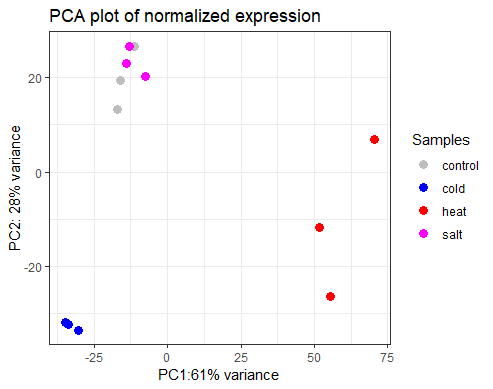


**SFigure 2.** Principle Component Analysis (PCA) of normalized expression in DH1 plants grown in the control, cold, heat and salt stress.


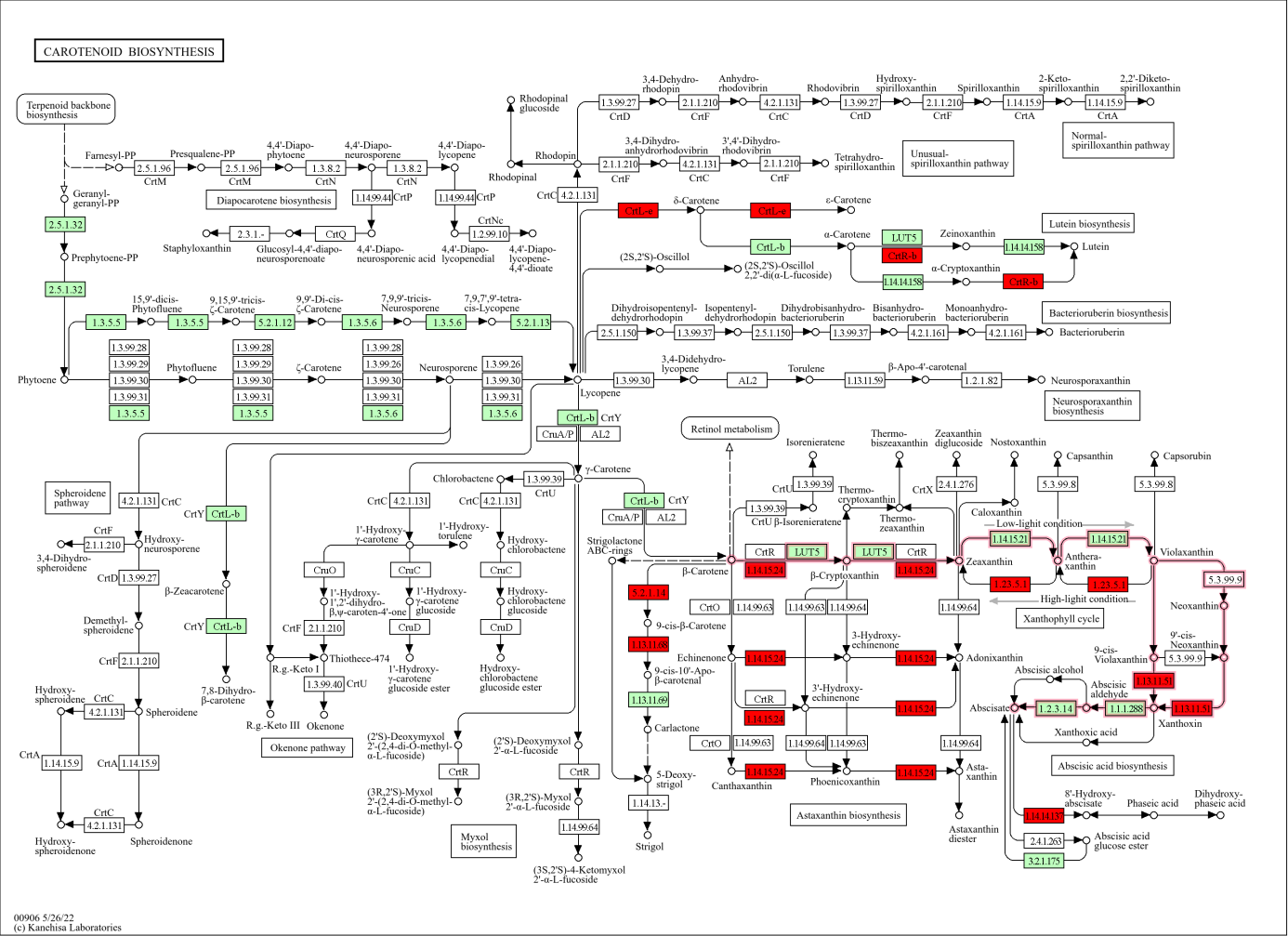


# SFigure 3. Carotenoid biosynthesis pathway (ath00906). Carrot genes with experimentally identified LHY binding sites in their promoters are shown in red. [M00372](https://www.genome.jp/module/ath_M00372) Abscisic acid biosynthesis module is highlighted by red arrows.

**
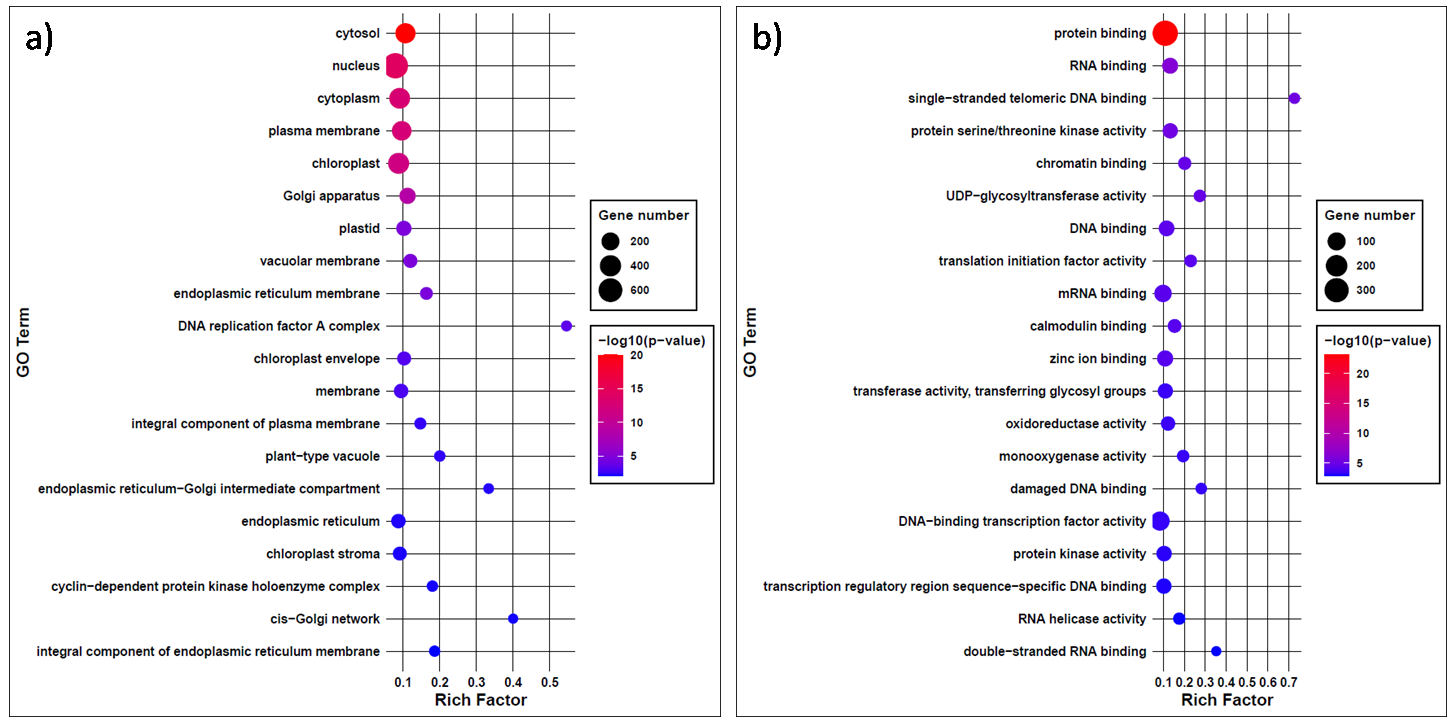
**

**SFigure 4.** Top 20 GO terms in cellular component (a) and molecular function (b) categories for genes to which LHY binds in the promoter region.

**
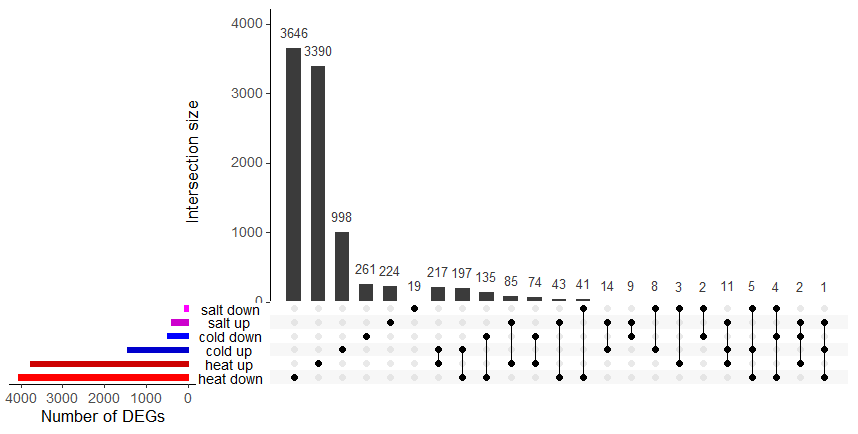
**

**SFigure 5.** Summary of carrot upregulated (dark shade) and downregulated (pale shade) genes under salt (magenta), cold (blue), and heat (red) stress.

**
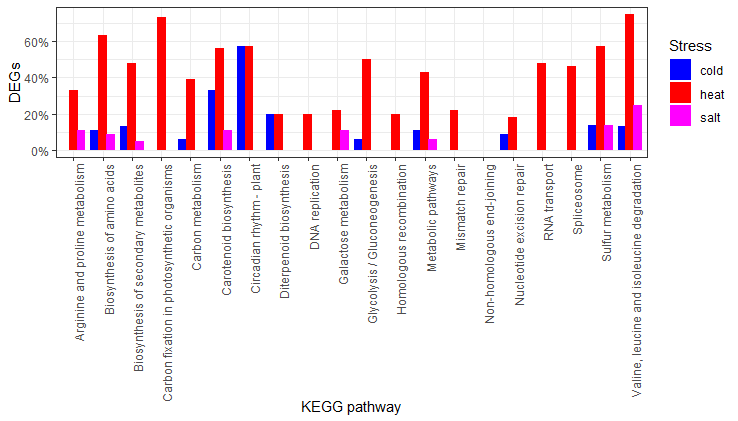
SFigure 6.** Fraction of DEGs in top 20 KEGG pathways to which LHY binds in the promoter region.

**
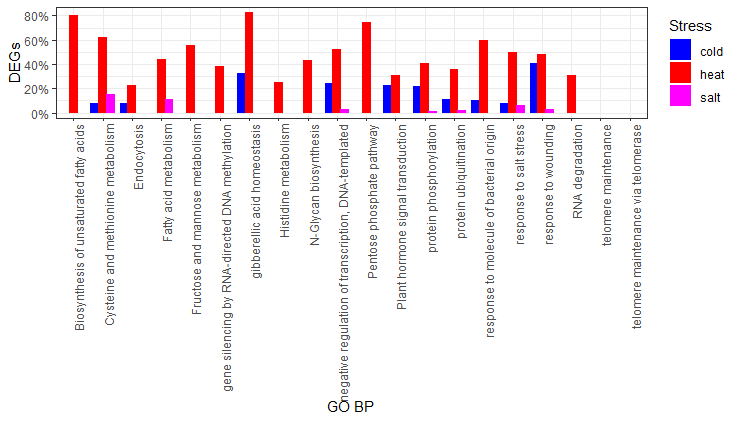
**

**SFigure 7.** Fraction of DEGs in top 20 GO terms in biological function category to which LHY binds in the promoter region.

**
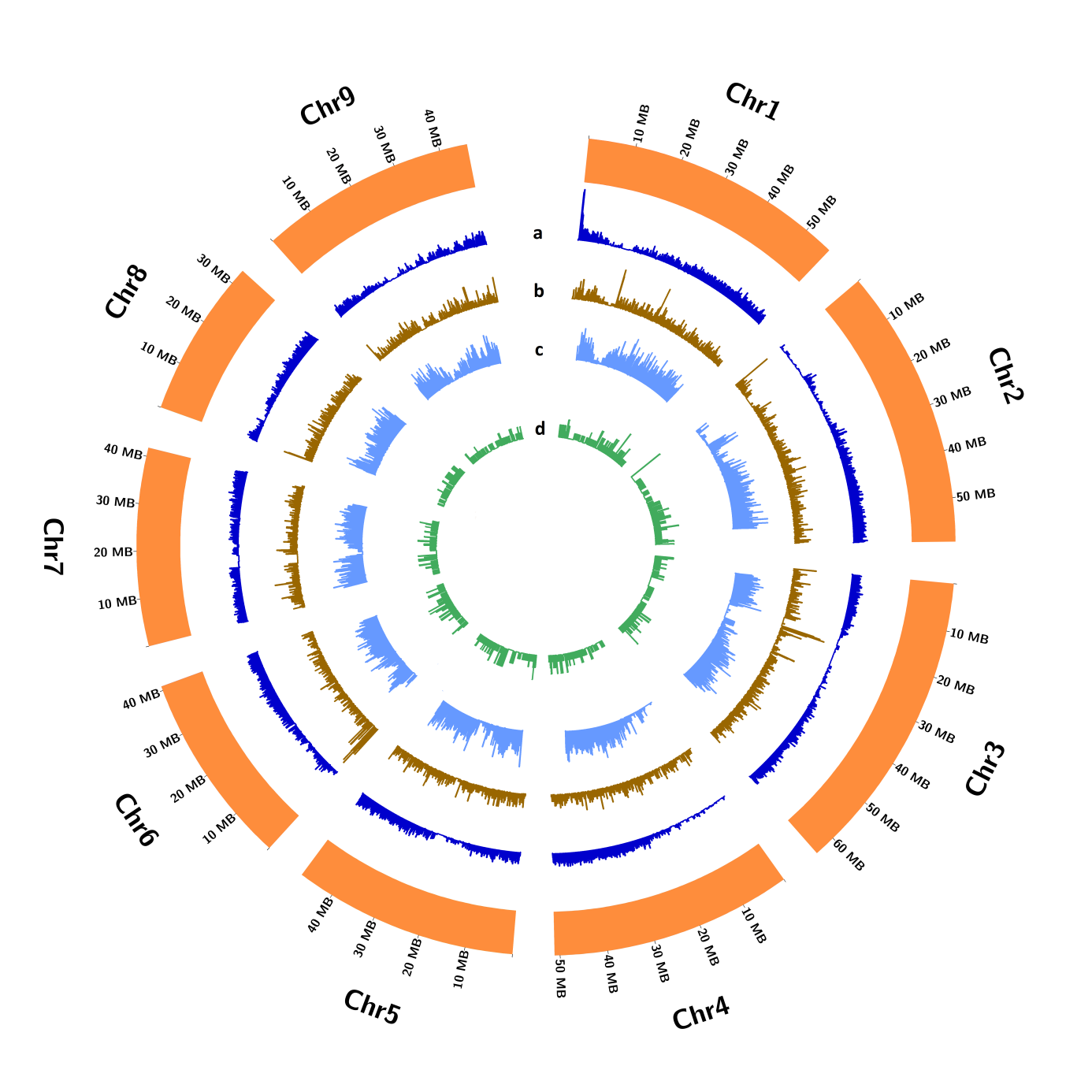
**

**SFigure 8.** Genomic distribution of genes (a), DcLHY binding sites (b), MITEs (c), and *DcTourist_15* copies (d) in the carrot genome.

**
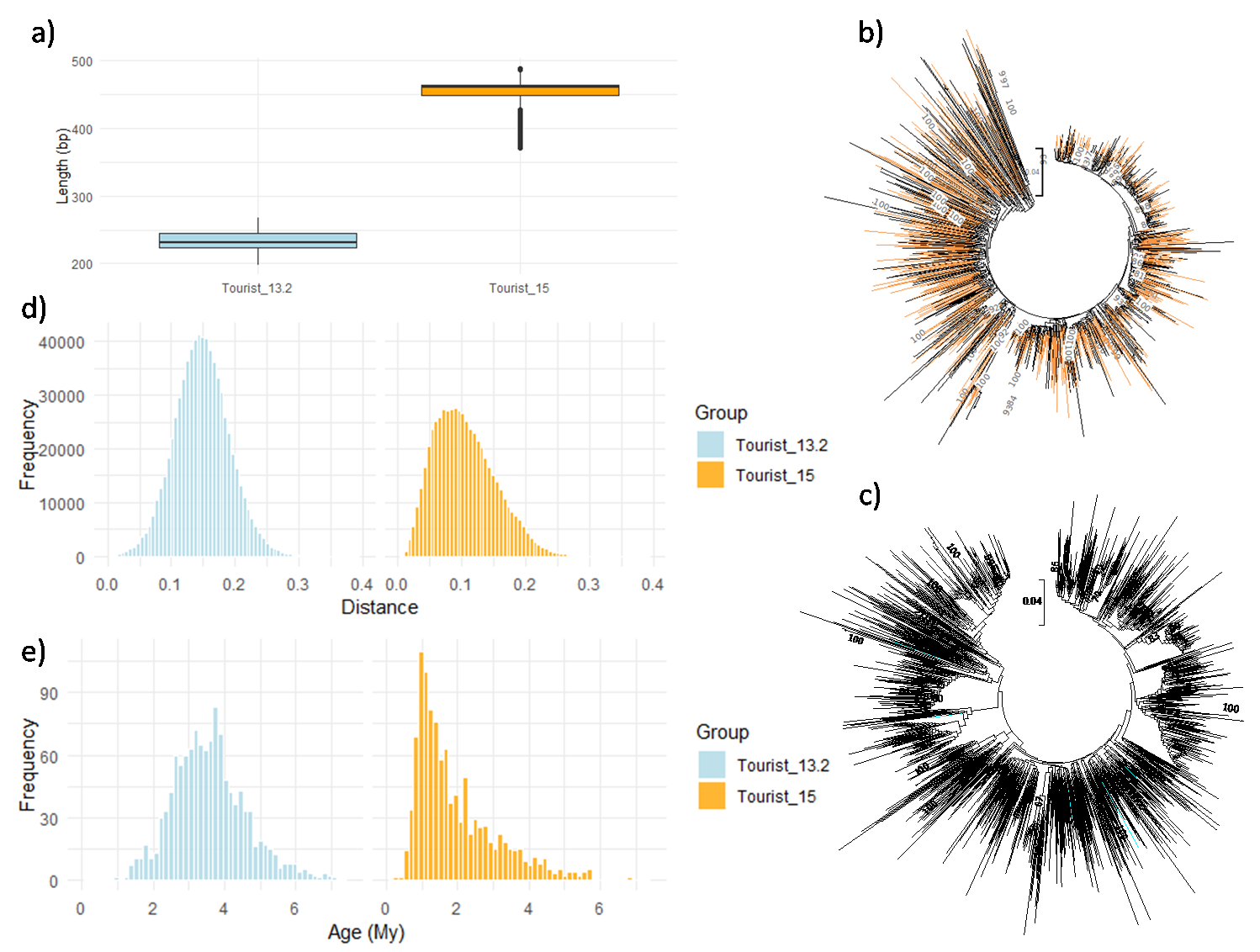
**

**SFigure 9.** Characteristics of *DcTourist_15* and *DcTourist_13.2* families in the carrot genome. (a) Box plot showing copy length distribution; (b) NJ tree of *DcTourist_15*; (c) NJ tree of *DcTourist_13.2*. Branches representing copies located in regions bound by DcLHY are highlighted in orange and blue for *DcTourist_15* and *DcTourist_13.2*, respectively; (d) Distribution of pairwise genetic distances between copies; (e) Age distribution calculated based on distances from the consensus sequences.

**SFigure 10.** Localization of carrot MITEs in the genomic context, all *Tourist* elements and two *Tourist* families, *DcTourist_15* and *DcTourist_13.2*. Upstream and downstream regions are within 2kb of the nearest genes, while genic regions include both introns and cds.

**
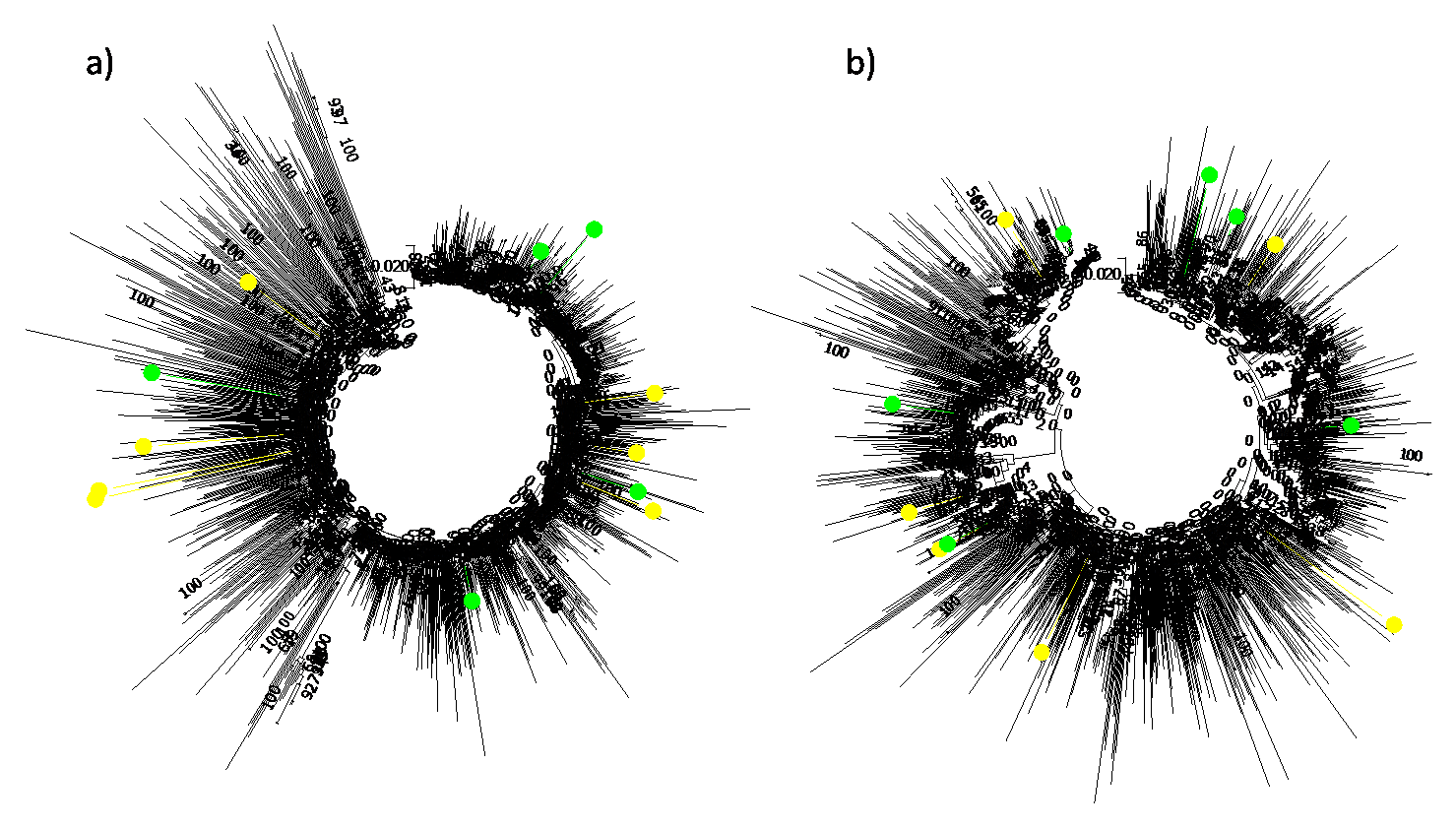
**

**SFigure 11.** Localization of *DcTourist_15* (a) and *DcTourist_13.2* (b) copies on a NJ tree, that were monomorphic (yellow) and polymorphic (green), as assayed by PCR.

**
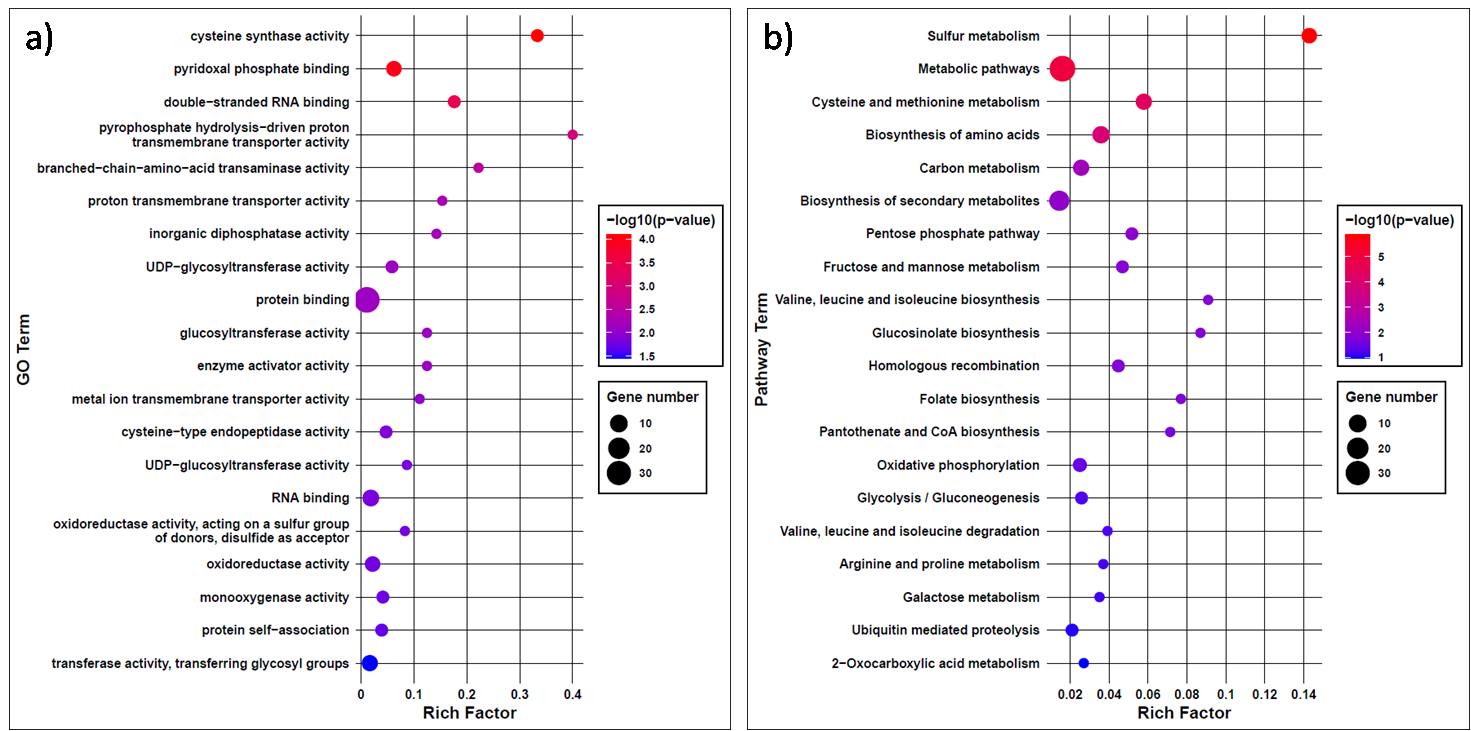
**

**SFigure 12.** Top 20 GO terms assigned to molecular function (MF) (a) and KEGG terms (b) for genes with DcLHY binding sites in their promoters overlapping with *DcTourist_15* copies.

**
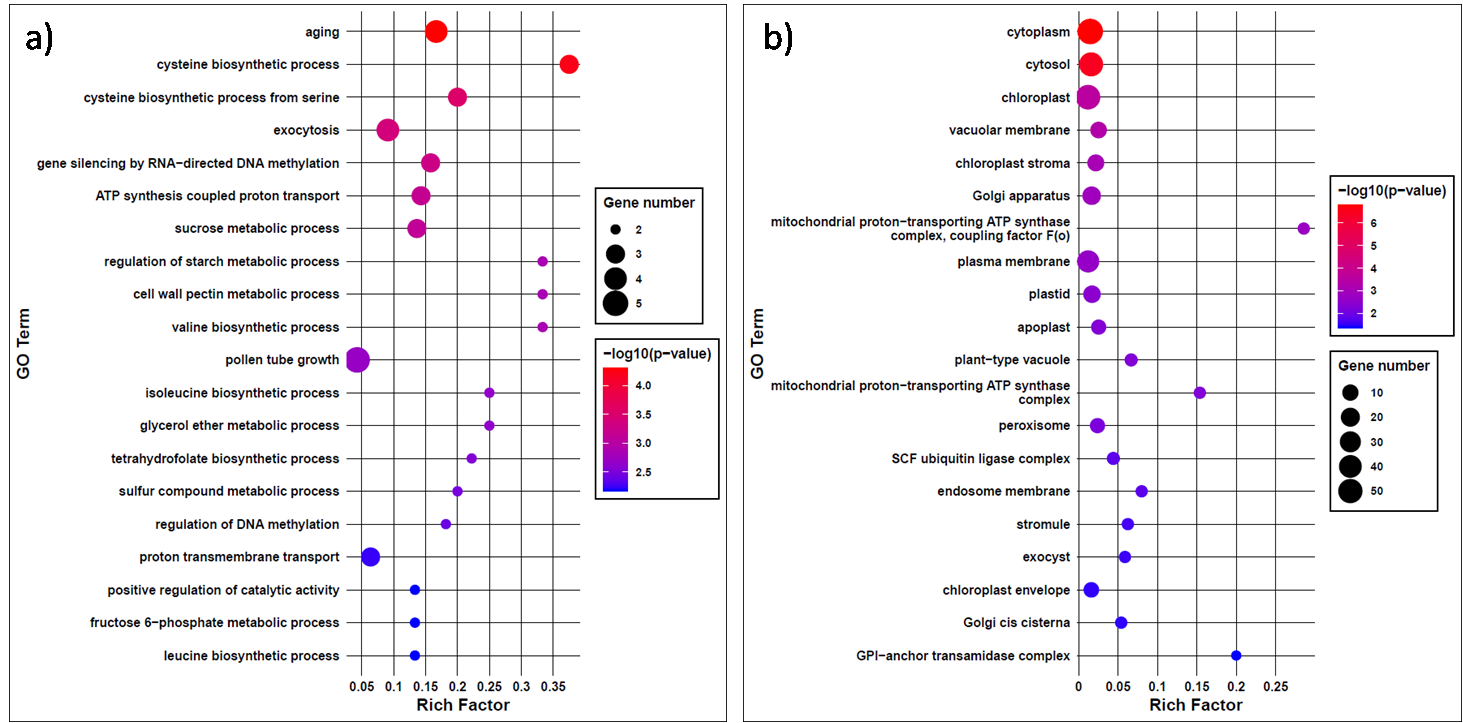
**

**SFigure 13.** Top 20 GO terms in biological process (BP) (a) and cellular component (CC) (b) categories of genes with LHY binding sites in their promoters overlapping with *DcTourist_15* copies.


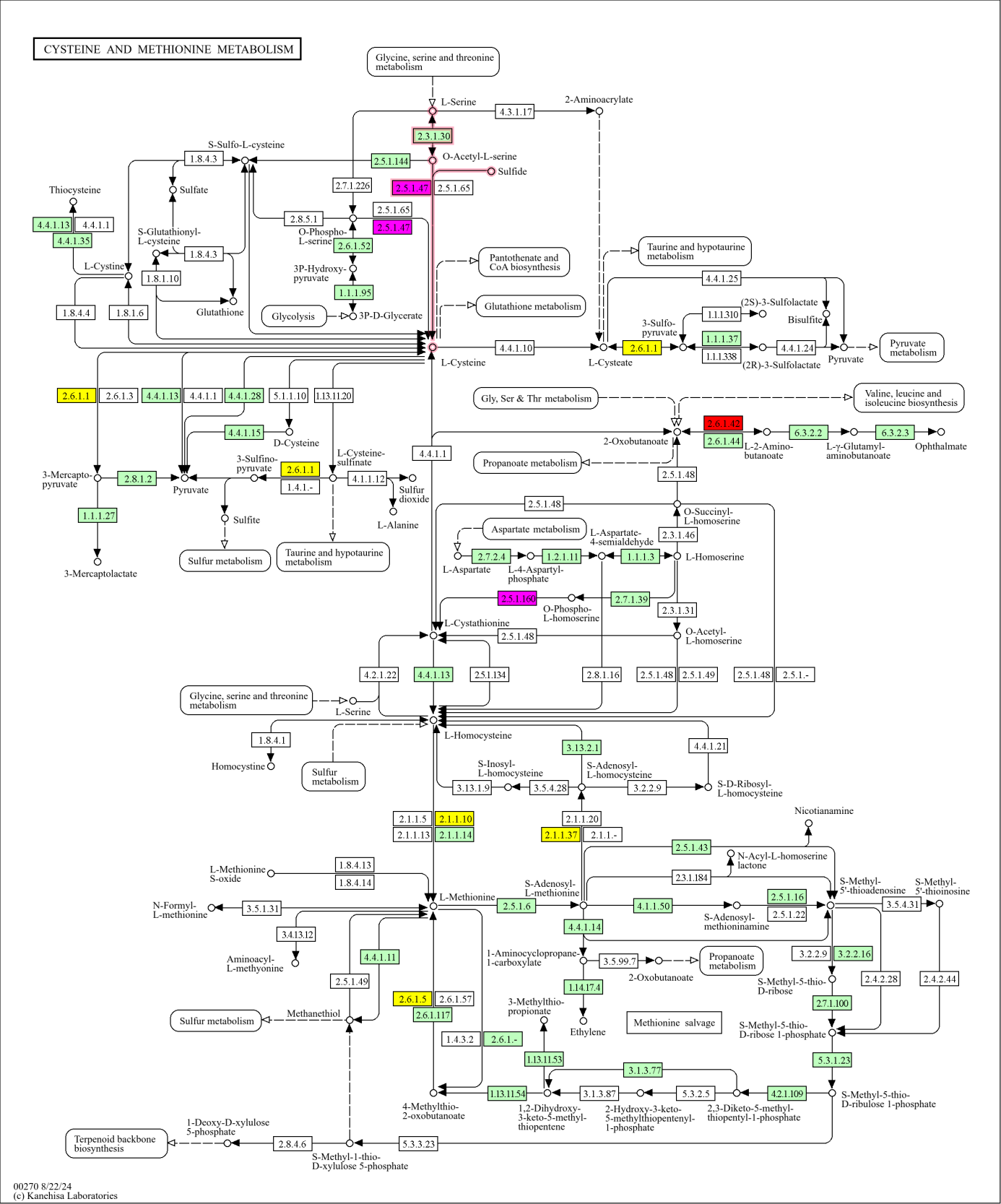


**SFigure 14.** Cysteine and methionine metabolism pathway (ath00270). Carrot genes with experimentally identified DcLHY binding sites in their promoters are shown in yellow, genes that are also associated with *DcTourist_15* insertions are shown in red, genes associated with *DcTourist_15* involved in sulfur metabolism are shown in purple. M00021 Cysteine biosynthesis module is highlighted by red arrows.


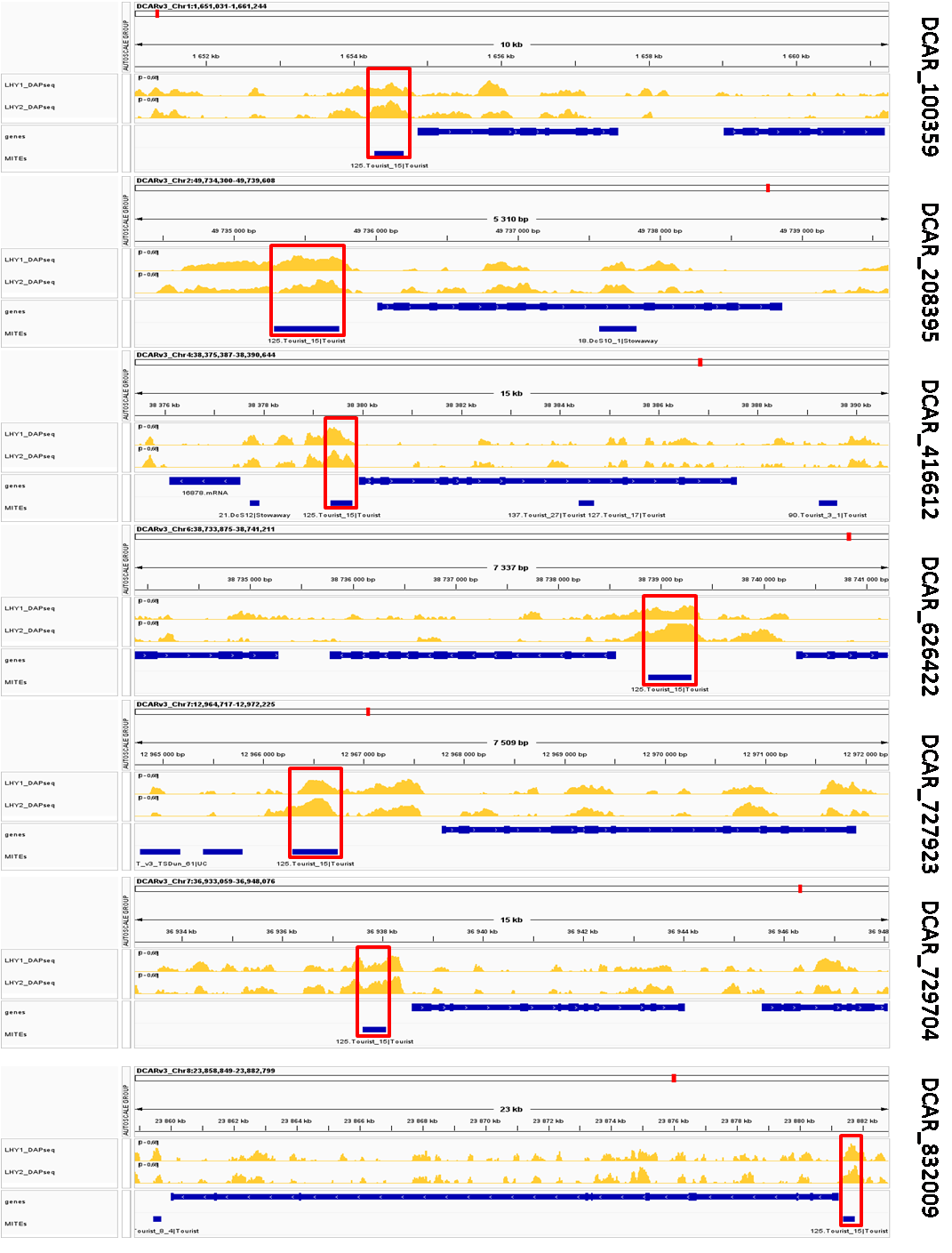


**SFigure 15.** Carrot genes involved in cysteine and methionine metabolism, with *DcTourist_15* insertions in promoters. Yellow tracks show DAP-seq coverage (two technical replicates). *DcTourist_15* insertions are highlighted by red frames.

**SFigure 16.** PCoA plot showing relationships among the ten carrot breeding lines evaluated using *DcS*-ILP markers (Stelmach et al. 2017).


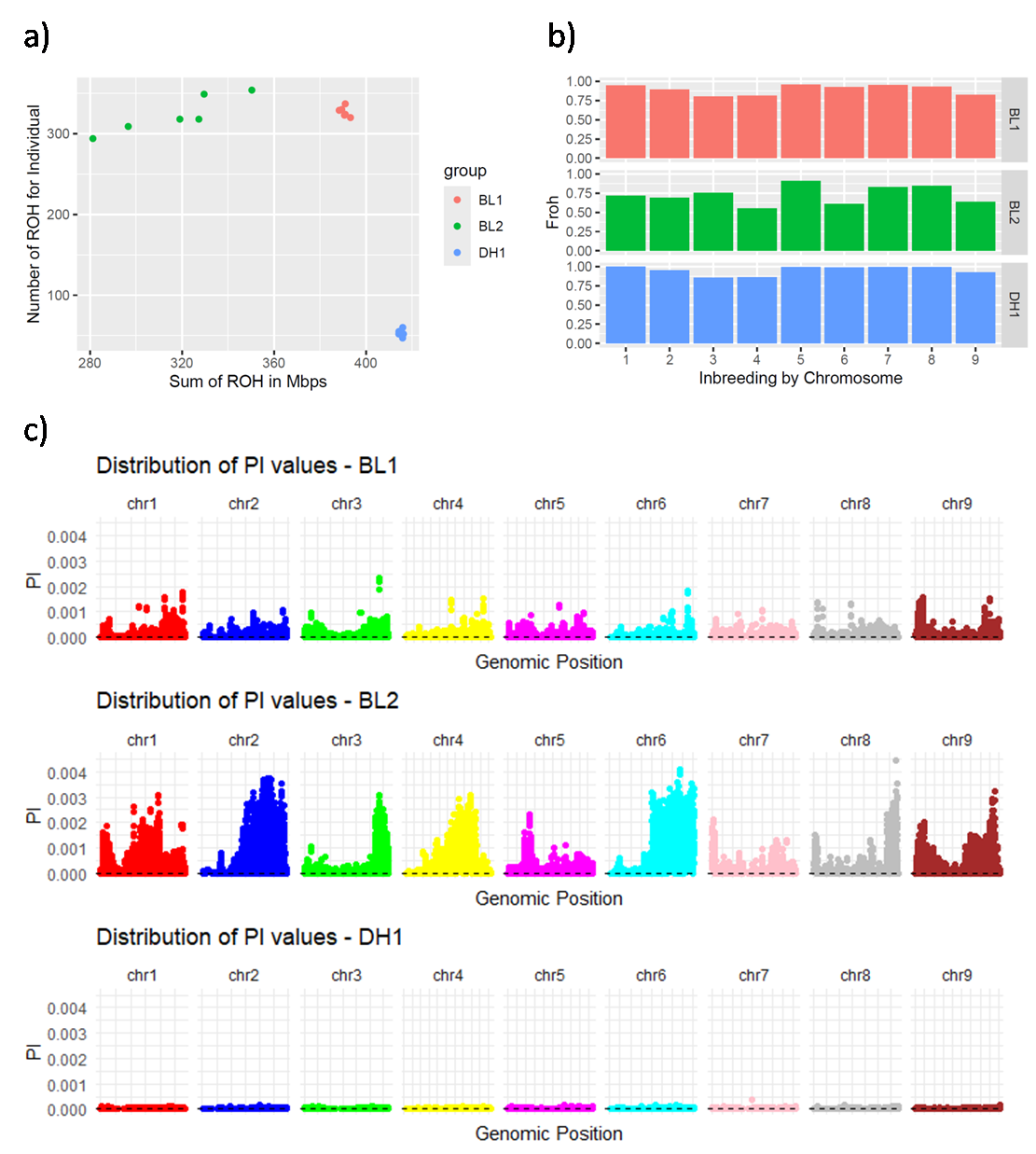


**SFigure 17**. Homozygosity of plants from two carrot breeding lines 493B (BL1) and Nh2168B (BL2) and the reference line DH1. Number and proportion of runs of homozygosity (ROH) in the genome (a), ROH-based inbreeding coefficient (b), and nucleotide diversity (c) based on SNPs called from RNAseq reads.


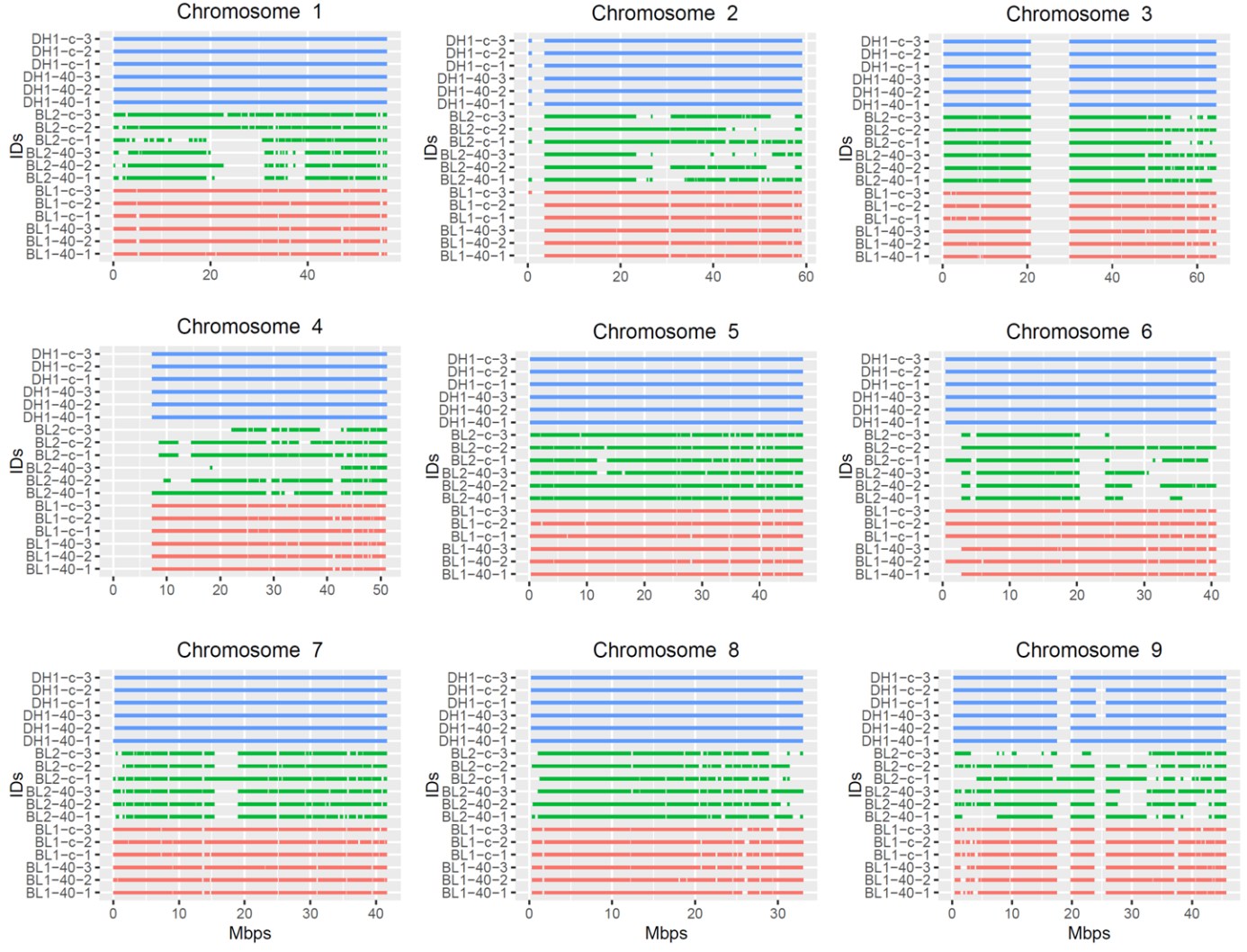


**SFigure 18.** Distribution of long runs of homozygosity (LROH) detected from RNAseq reads across chromosomes in two carrot breeding lines 493B (BL1 - red), NH2168B (BL2 - green) and the reference line DH1 (blue).


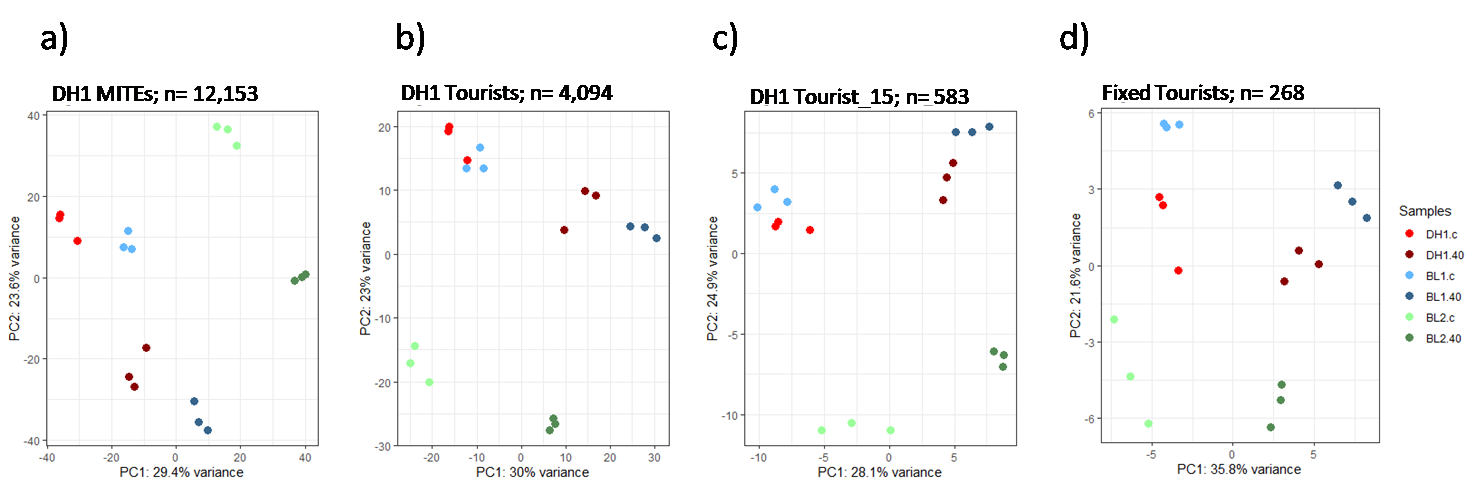


**SFigure 19.** PCA on normalized expression of carrot genes in DH1 and two breeding lines grown under heat stress and in control conditions containing MITE insertions in the DH1 reference genome (a); reference *Tourist* insertions (b); reference *DcTourist_15* insertions (c); and *Tourist* insertions shared among the three lines (d).


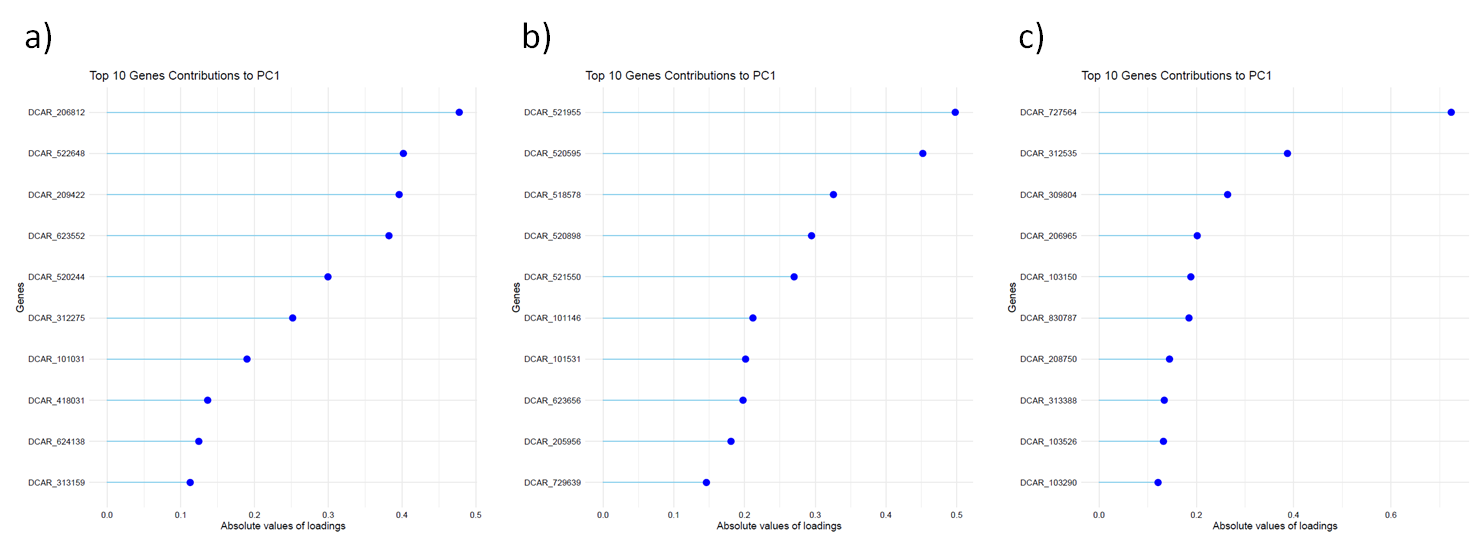


**SFigure 20.** Contribution of top 10 genes to grouping in the PCA plot obtained based on normalized expression of carrot genes in DH1 and two breeding lines grown under heat stress and in control conditions containing *DcTourist_15* insertions shared among the three lines (a); *DcTourist_15* insertions present in DH1 and 493B and absent in Nh2168B (b); *DcTourist_15* insertions present in DH1 and Nh2168B and absent in 493B (c).


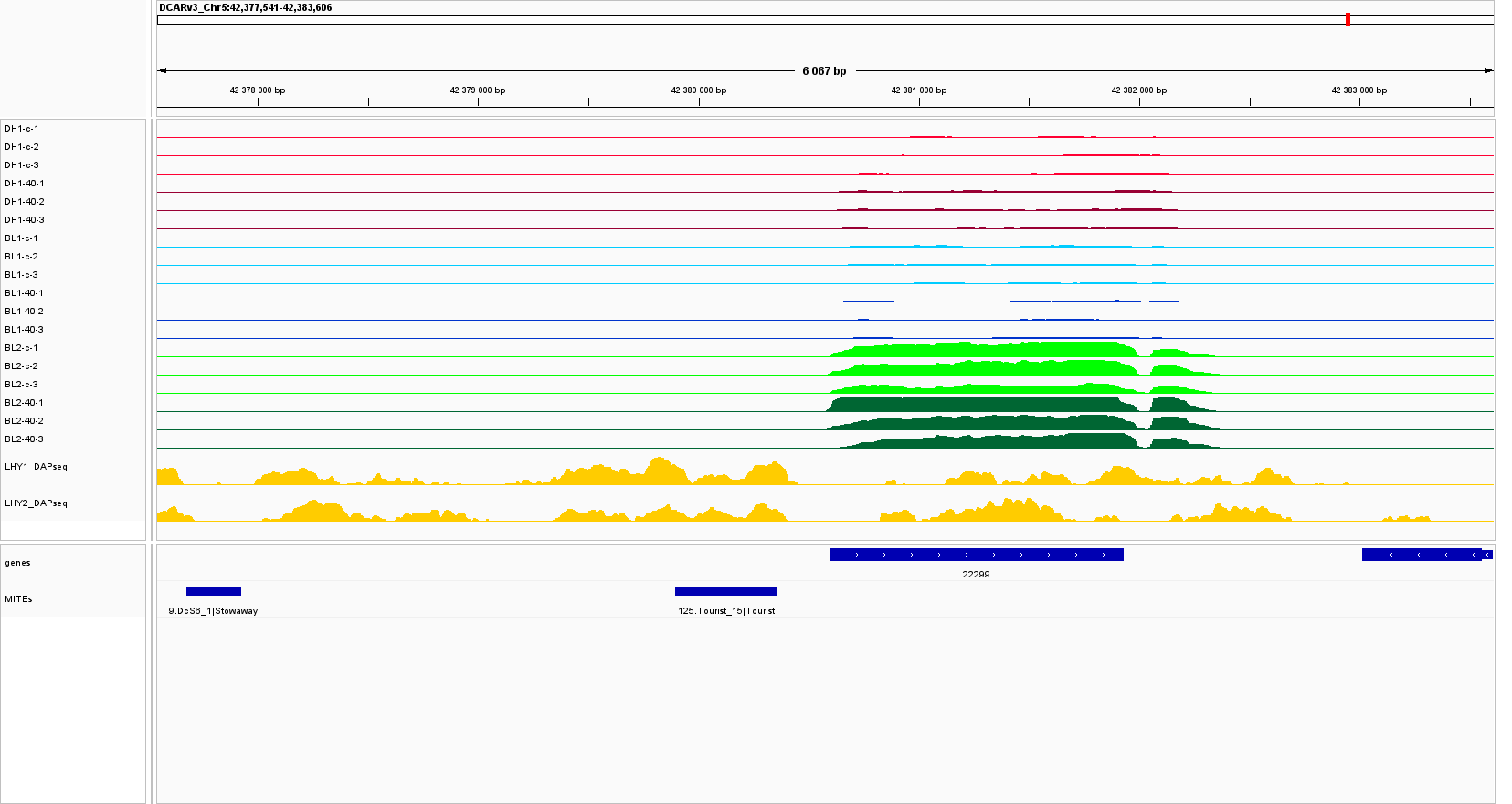


**SFigure 21.** DCAR_521955 gene, with a *DcTourist_15* copy in the promoter, showing lower expression as compared to the variant without insertion and a slight upregulation under heat. Red, blue, and green tracks show expression levels in DH1, 493B (BL1) and Nh2168B (BL2), respectively, in control (pale) and heat (dark). Yellow tracks show genome coverage by DAPseq reads (two technical replicates).


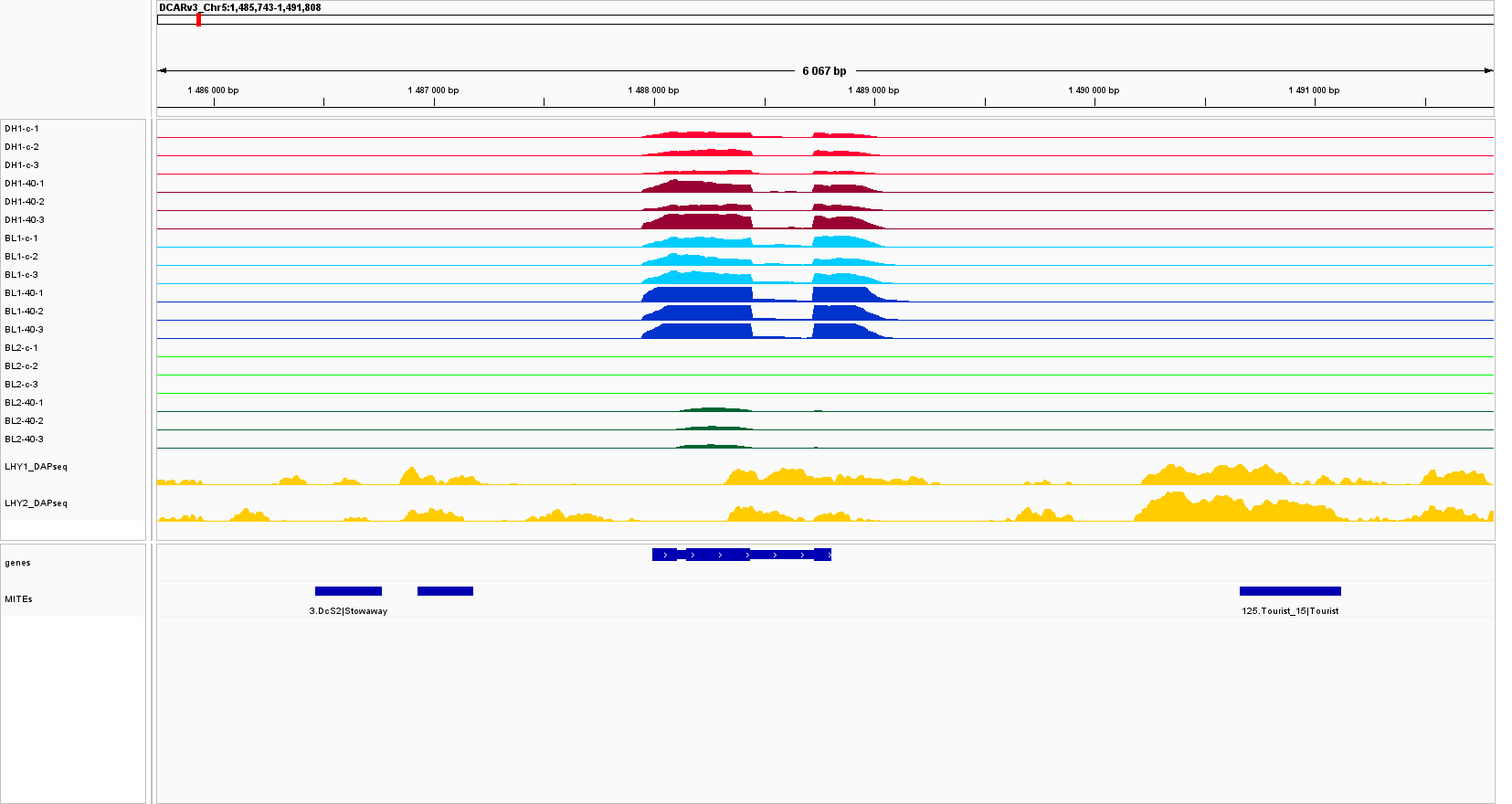


**SFigure 22.** DCAR_518578 gene, with a *DcTourist_15* copy inserted downstream, not expressed in the variant without insertion and upregulated under heat. Red, blue, and green tracks show expression levels in DH1, 493B (BL1) and Nh2168B (BL2), respectively, in control (pale) and heat (dark). Yellow tracks show genome coverage by DAPseq reads (two technical replicates).


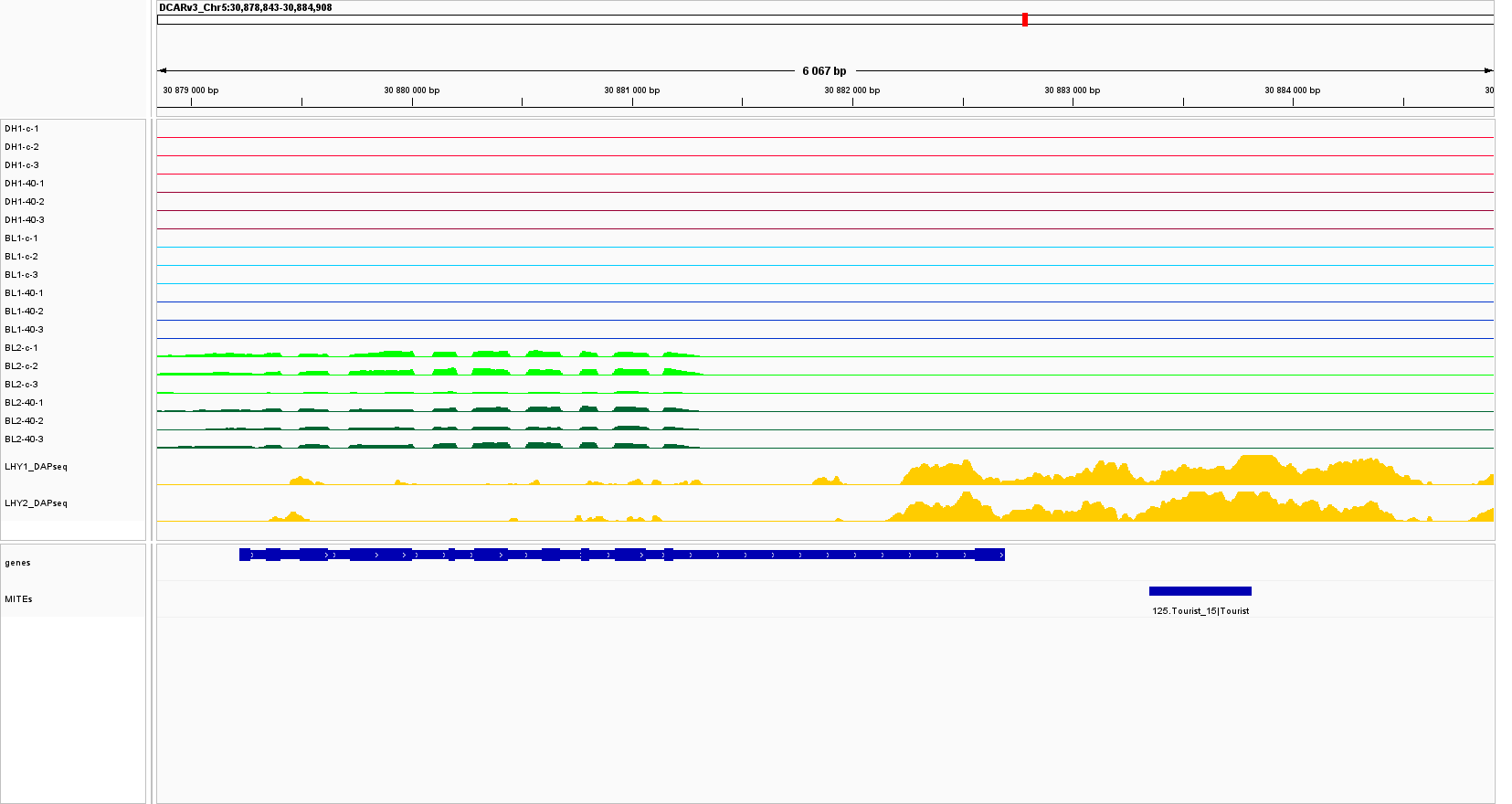


**SFigure 23.** DCAR_520595 gene, with a *DcTourist_15* copy inserted downstream, silenced in the variant carrying the insertion. Red, blue, and green tracks show expression levels in DH1, 493B (BL1) and Nh2168B (BL2), respectively, in control (pale) and heat (dark). Yellow tracks show genome coverage by DAPseq reads (two technical replicates).
